# Standardized *Bacopa monnieri* Extract Ameliorates Learning and Memory Impairments through Synaptic Protein, Neurogranin, Pro-and Mature BDNF Signaling, and HPA Axis in Prenatally Stressed Rat Offspring

**DOI:** 10.3390/antiox9121229

**Published:** 2020-12-04

**Authors:** Karunanithi Sivasangari, Koilmani Emmanuvel Rajan

**Affiliations:** Department of Animal Science, Behavioural Neuroscience Laboratory, Bharathidasan University, Tiruchirappalli 620024, India; sangarik@bdu.ac.in

**Keywords:** prenatal stress, *Bacopa monnieri*, caspase-3, synaptophysin, *N*-methyl-D-aspartate receptor (NMDAR), brain-derived neurotropic factor (BDNF)

## Abstract

Prenatal stress (PNS) influences offspring neurodevelopment, inducing anxiety-like behavior and memory deficits. We investigated whether pretreatment of *Bacopa monnieri* extract (CDRI-08/BME) ameliorates PNS-induced changes in signaling molecules, and changes in the behavior of Wistar rat offspring. Pregnant rats were randomly assigned into control (CON)/prenatal stress (PNS)/PNS and exposed to BME treatment (PNS + BME). Dams were exposed to stress by placing them in a social defeat cage, where they observed social defeat from gestational day (GD)-16–18. Pregnant rats in the PNS + BME group were given BME treatment from GD-10 to their offspring’s postnatal day (PND)-23, and to their offspring from PND-15 to -30. PNS led to anxiety-like behavior; impaired memory; increased the level of corticosterone (CORT), adrenocorticotropic hormone, glucocorticoid receptor, pro-apoptotic Casepase-3, and 5-HT_2C_ receptor; decreased anti-apoptotic Bcl-2, synaptic proteins (synaptophysin, synaptotagmin-1), 5-HT_1A,_ receptor, phosphorylation of calmodulin-dependent protein kinase II/neurogranin, *N*-methyl-D-aspartate receptors (2A,2B), postsynaptic density protein 95; and conversion of pro and mature brain derived neurotropic factor in their offspring. The antioxidant property of BME possibly inhibiting the PNS-induced changes in observed molecules, anxiety-like behavior, and memory deficits. The observed results suggest that pretreatment of BME could be an effective coping strategy to prevent PNS-induced behavioral impairments in their offspring.

## 1. Introduction

Animal model and clinical studies have demonstrated the effect of prenatal stress (PNS) and exposure to antidepressants from the perspective of the offspring [1,2,3,4]. Parallel studies have documented that PNS-induced behavioral changes were associated with changes in adrenocorticotropic hormone (ACTH), corticosterone (CORT) and its receptor (glucocorticoid receptor; GR), and the monoaminergic system [5,6,7]. The hyperactivity of the hypothalamic–pituitary–adrenal (HPA) axis led to the accumulation of corticosterone, which triggered the release of excitatory neurotransmitter (glutamate, Glu) [8]. The level of Glu increased in the synaptic cleft by stress, which caused learning and memory impairment through neuronal toxicity and degeneration [9] and could be linked with the regulation of pro-apoptotic caspase-3 and anti-apoptotic Bcl-2 [10,11]. Interestingly, earlier studies have reported that the interaction of corticotrophin-releasing hormone (CRH) with serotonergic neurons possibly interacted and regulated the release of serotonin (5-hydroxytryptamine, 5-HT) and in turn, 5-HT signals regulated the HPA axis function by regulating the level of CRH [12,13].

Dysregulation of the HPA axis has been known to alter the serotonergic system, thus, synaptic proteins (synaptophysin (SYP), synaptotagmin-1 (SYT-1)) and 5-HT receptors were also altered [14,15]. Furthermore, the synaptic transmission, finely tuned by interaction between the presynaptic and postsynaptic proteins and among the presynaptic proteins [16], regulated behavior [17]. In addition, the 5-HT_1A_/5-HT_2C_ receptor has been known to critically regulate anxiety/anxiolytic behavior [18,19,20]. Activation/inhibition of 5-HT receptor critically regulate the signaling pathway mediated by phosphorylation of calmodulin-dependent protein-kinase II (CaMKII) and *N*-methyl-D-asparate (NMDA) receptors [21,22]. Earlier studies have shown that activation of CaMKII led to formation of a complex with NMDA receptors (NR2A, NR2B), which was critical for stable long-term potentiation (LTP) [23,24]. Furthermore, this complex regulates the localization of postsynaptic density protein 95 (PSD-95) in the spine, and its interactions with brain-derived neurotrophic factor (BDNF) [24,25]. Precursor and mature BDNF, and its downstream signaling molecules are known to influence synaptic plasticity and have been linked with behavioral disorders [26,27].

In addition, in the Indian traditional system of Ayurvedic medicine, *Bacopa monnieri* (Linn.) has been used as a nootropic agent in Ayurvedic herbal formulations and has been classified as a medhyarasayana, a drug used to cure mental disorders. The characterization and structural elucidation of *B. monnieri* extract reveals the presence of bacosides [bacoside A (bacogenins A1, A2, A3, and A4), bacoside B, bacopacoside II, bacopasaponin X, and bacopasaponin C], which possibly hold the property to improve cognitive function [28,29,30]. Studies in animal models of depression and anxiety have shown that *B. monnieri* extract treatment reduced anxiety-like behaviors [31,32]. In addition, *B. monnieri* extract (CDRI-08) supplementation reduced anxiety and depression in elderly people [33,34]. Homeostatic adaptation of hormones and neurotransmitters mediate stress adaptation, in which a balancing act of oxidants and antioxidants can play a crucial role [35]. Dietary supplement with antioxidants has been shown to be as effective for protecting against stress-induced abnormality [36].

In this study, we hypothesized that, in our PNS model, exposure to *Bacopa monnieri* extract (CDRI-08) may have a resilience effect on PNS outcome in offspring. To test this, we examined behaviors in an experimental group of adolescent rats and further estimated the level of CORT, ACTH, expression of GR, caspase-3, synaptic proteins (SYP, SYT-1), 5-HT receptors (5-HT_1A/2C_), CaMKII/p-CaMKII, NR2A/B receptors, PSD-95, as well as pro-and mature BDNF, in their offspring.

## 2. Materials and Methods

### 2.1. Bacopa monnieri Extract

The extract was prepared from the whole plant of *Bacopa monnieri* (CDRI-08, referred to as BME in this articles) using ethanol (70%), and then the solvent was removed. Then, the soluble was concentrated with 95% ethanol, dried in vacuo, and macerated with acetone, obtaining the free flowing powder. The bacoside (bacopaside A3, bacopacoside II, bacopasaponin X, and bacopasaponin C) enriched extract was obtained from Dr. Hemant Singh as a generous gift, Lumen marketing company, Chennai, India (batch# C15030294). The high performance liquid chromatography (HPLC) chromatogram of the extract is provided as Supplementary Data-1 [37].

### 2.2. Animal Design

The experimental timeline is depicted in Supplementary Figure S1 in Supplementary Materials. Female Wistar rats (*Rattus norvegicus,* 180–250 g) were selected by continuously monitored their estrous cycle and the distribution of different stages during the estrous cycle [38]. Selected Dams were individually housed with one sexually experienced male for mating. Gestational day (GD)-0 was designated by the presence of sperms in vaginal lavage sample and pregnant rats were housed individually in standard laboratory cage under a controlled environment (24 ± 2 °C, 12 h light-dark cycle) with ad libitum water and food. Pregnant rats were randomly assigned into the following three groups: (i) control (CON); (ii) prenatal stress (PNS, received 0.5% gum acacia treatment (per-orally, p.o.)); and (iii) prenatal stress + exposed to BME (PNS + BME 80 mg/kg + 0.5% gum acacia (p.o.)) treatment. In order to select the optimum dose of BME, different concentrations (40, 60, 80, 100 mg/kg) of BME were provided to the individual dams, and then they were tested using a behavioral task [36]. Freshly prepared BME aqueous suspension (BME + gum acacia in double distilled water) or gum acacia aqueous suspension (gum acacia in double distilled water) were treated orally to the dams each day (10.00 to 11.00 h) from GD-10 to their pup’s postnatal day (PND)-23 except on the day of parturition (≈8 h after parturition), and to the pups from PND-15 to -30. The day of parturition was noted as PND-0 and the male pups were separated on PND-24 from their dam and housed (2–3/cage) in different cages [39]. Two or three male pups were randomly selected from each dam (three dam for each group) for different experimental groups (i.e., control (*n* = 7), PNS (*n* = 6), and PNS + BME (*n* = 6)), and selected pups were subjected to the behavioral test on PND-31/-32. Throughout the study, care was taken to minimize the handling stress by partially replacing the bedding material periodically to ensure the home cage odor, and minimally disrupt the nests. All the experimental group rats were handled minimally to avoid handling effects. The project was recommended by the Institutional Animal Ethics Committees of Bharathidasan University, Tiruchirappalli, India (approval no. BDU/IAEC/P22/2018 dated 7 August 2018) and approved by the Committee for the Purpose of Control and Supervision of Experiments on Animals (CPCSEA) (reg. no. 418/GO/Re/S/01/CPCSEA dated 27 April 2018). All the experiments were conducted in compliance with the guidelines laid down by CPCSEA, Government of India, India.

### 2.3. Chemicals

All chemicals used in this study were of analytical grade.

### 2.4. Prenatal Stress

To induce prenatal stress (PNS), the pregnant rats were allowed to observe social defeat (SDO) from GD-16 to GD-18, in a specially designed social defeat cage (Supplementary Figure S2 in Supplementary Materials) for 10 min/day, following the procedure modified from Lee et al. [40]. The social defeat cage consisted of three chambers (observer chamber (OC), intruder chamber (IC), resident chamber (RC)) with equal size (30 × 30 × 30 cm). The RC was connected with a standard laboratory cage through a transparent plastic pipe (60 cm in length and 10.5 cm in diameter), the OC was permanently partitioned with wire mesh, and the RC and IC were partitioned with sliding wire mesh doors, which facilitated the aggressive interaction of the resident male and intruder male. Ten days before the experiment, the aggressor (senescence male rat) was housed in the standard laboratory cage which was connected to the RC and allowed the aggressor free access to the RC. First, the intruder (six-month-old male rat) was introduced into the IC, and after five minutes, the sliding wire mesh door was opened and the intruder was allowed to interact (10 min/day) with the aggressor in the RC. After the aggressive interaction the intruder was transferred to the home cage. The control group pregnant rats were allowed to explore the OC in the absence of aggressor and intruder from GD-16 to GD-18. The PNS and PNS + BME group were allowed to observe the social defeat (GD-16 to GD-18) from the OC.

### 2.5. Behavioral Test

#### 2.5.1. Elevated Plus Maze (EPM)

Experimental group pups were subjected to the elevated plus maze (EPM) test from PND-31 to PND-32. The EPM test was used to identify the anxiety-like behavior in rats; the two closed arm (30 × 5 × 15 cm) and two open arms (30 × 5 × 0.25 cm) were connected to the central platform and were 50 cm above the floor level [41]. Three hours before the behavioral test, animals were shifted to the behavioral testing room and kept undisturbed. Individually, test animals were placed in the central square of the EPM, facing towards an open arm and they were allowed to explore for five minutes. Individuals’ behavior was video recorded for analysis. The entire behavior test was conducted under bright illumination (30 W). The apparatus was cleaned with 75% ethanol after every trial to remove olfactory cues.

#### 2.5.2. Y-Maze Test

Individual rat’s spontaneous alternation behavior was tested using a Y-maze apparatus. The Y-shaped apparatus was constructed with three horizontal arms to connect symmetrically at a 120° angle (40 × 5 × 15 cm) [42]. The behavioral test was conducted in a behavioral room which was illuminated with a 100 W bulb. On PND-30, the experimental animal was placed in the start arm end and allowed to explore the maze for 15 min. The next day, the B arm was closed, and the reward was placed in the C arm to ensure that the C arm was their first choice for 10 min. On PND-32, the testing was conducted for 10 min by allowing the rat to investigate all three arms, in which the closed arm was presented as a novel arm and the reward was placed in the C arm. An entry was calculated when a rat placed all four paws in an arm. Pure chocolate was provided as the reward without nuts or any other compounds. Training and testing were conducted as 2 trials/day with four-hour interval. On the day of testing, individuals’ behavioral profiles, such as total number of arm entries and spontaneous alternation (number of traits), were recorded and analyzed. As triplet sets (i.e., ABC/BCAo/CAB but not ABA/BCB/CAC) the entries were designated to calculate spontaneous alternation. The alternations (% alternations) were calculated as spontaneous alternations/(total number of arm entries − 2) × 100.

### 2.6. Hormone Assay

To estimate the level of corticosterone (CORT) and adrenocorticotropic hormone (ACTH), blood samples were collected from rats representing each group (control, PNS, PNS + BME, *n* = 6) into a tube with anticoagulant (sodium citrate, 0.25 mL of 3.8% sodium per 2.0 mL of blood). Plasma was separated by centrifuging at 1800× *g* for 10 min [43] and stored at −20 °C. The CORT and ACTH levels in plasma were estimated by using an ELISA kit (ALPCO Diagnostics, Salem, NH, USA).

### 2.7. Total RNA Isolation

Animals (*n* = 6) representing each group were sacrificed after the behavioral test. The whole brain was dissected out and placed on an ice-cold petri dish. The amygdala tissue was carefully dissected from the brain slice, as reported earlier [44]. One portion of the dissected amygdale was used to isolate RNA and the other side for isolation of protein. Total RNA was isolated by using TRI Reagent (cat. # FATRR 001; Favogen Biotech Corp, Pingtung, Taiwan) and stored at −80 °C. Total RNA (2 µg) was used to synthesis cDNA (cat.# 170-8891; Iscript^TM^ cDNA synthesis kit, Bio-Rad laboratories Inc., Hercules, CA, USA).

### 2.8. Protein Isolation

Amygdala tissue obtained from the experimental groups were homogenized in ice-cold lysis buffer (150 mM NaCl, 50 mM Tris-Hcl pH 7.5, 5 mM EDTA, 0.1% *v/v* NP-40, 1.0 mM DTT, 0.2 mM sodium orthovanadate, 0.23 mM PMSF) containing protease inhibitor (4 μL/mL) (Sigma-Aldrich, Saint Louis, MO, USA). The homogenate was incubated in ice for 30 min, followed by centrifuged at 4 °C (10,000× *g*) for 30 min. The supernatants were collected in anew tube and again centrifuged at 4 °C (12,000× *g*) for 15 min, after being extracted, the samples as aliquots were stored at −80 °C [36,45,46,47]. Protein was quantified at 595 nm by Bradford method [48] (cat. #5000006; Bio-Rad Protein Assay kit, Bio-Rad laboratories Inc., Hercules, CA, USA) using a Biophotometer Plus (Eppendorf Inc., Hamburg, Germany).

### 2.9. Quantitative Real-Time PCR (qRT-PCR)

Total reaction volume (10 µL) contains an aliquot of real-time mixture (SYBR green super mix, Bio-Rad laboratories Inc.) with cDNA (0.2 µg) and specific primers (100 pmoles) [36]. Specific primers were used to estimate the expression of 5-HT_1A_ (accession number NM_012585.1; forward 5′-GACTACGTGAACAAGAGGAC-3′ and reverse 5′-TATAGAAAGCGCCGAAAGTG-3′), 5-HT_2C_ and (accession number NM_012765.3; forward 5′-AAACTGCACAATGCTACCAA-3′ and reverse 5′-TGATGGACGCAGTTGAAAAT-3′) normalized with internal control and glyceroldehydes -3-phosphate dehydragenase (GAPDH) (accession number NM_017008.4; forward 5′-AACATCAT CCCTGCATCCAC-3′ and reverse 5′-AGGAACACGGAAGGCCATGC-3′). The reaction starts with 92 °C initial denaturation (3 min), and then 40 cycles of denaturation at 92 °C (5 s), annealing (5 s) (5-HT_1A_ (52.4 °C), 5-HT_2C_ (59.7 °C), GAPDH (56 °C)), extension at 72 °C (5 s), and final extension at 72 °C (10 min). Single specific PCR product amplification was confirmed by observing the dissociation curve followed by melting curve analysis (CFX-96 Touch Real-Time PCR detection system; CFX manager version 2 software; Bio-Rad laboratories Inc., Hercules, CA, USA) [49,50]. The data obtained from triplicate were normalized with internal control and presented as the mean fold change relative to the control group.

### 2.10. Western Blot Analysis

An equal concentration of total protein (80 µg) separated on polyacrylamide gel (PAGE 10%) was mixed with a buffer (2% mercaptoethanol, 100% glycerol, 4% SDS, 125 mM Tris-HCL pH 6.8, 0.006% bromophenol blue) and boiled (2 min). Semi-dry Western blot apparatus (SD-20; Cleaver Scientific Ltd., Rugby, UK) was used to transfer the separated protein to the polyvinylidine difluride membrane (PVDF) (cat. #IPVH00010; Millipore, Burlington, MA, USA). The membranes were preblocked in Tris-buffered saline (TBS, 10 mM Tris-base pH 7.5 and 150 mM NaCl) containing Tween-20 (0.1%) and non-fat milk (5% for 2 h) at room temperature. Membranes were incubated with any one of the following primary antibodies for 12–15 h at 4 °C: rabbit-anti-GR (SC-1004, 1:1000); rabbit-anti-caspase-3 (SC-7148, 1:1000); rabbit-anti-Bcl2 (ab59348, 1:1000); rabbit-anti-SYP (M-04-1019, 1:10,000) (Millipore, Burlington, MA, USA); mouse anti-SYT1 (BD-610433, 1:4000)(BD Biosciences, San Jose, CA, USA); mouse anti-total-αCaMKII a (t-αCaMKII, SC-32288, 1:200); rabbit anti-phosphorylated αCaMKII (p-αCaMKII, Thr^286^ SC-12886-R, 1:200); rabbit anti-Ng (ab23570, 1:1000); rabbit anti-pNgantibody (ABN 426, 1:1000); rabbit polyclonal NR2A antibody (BT-AP02388, 1:2000); rabbit NR2B (BT-AP02389, 1:2000); rabbit anti-PSD-95 (SC-28941, 1:200); rabbit anti-pro-BDNF (SC-20981 (H-117), 1:500); rabbit anti-BDNF (SC-546 (N-20), 1:500).For the internal control rabbit polyclonal anti-rabbit-β-actin (SC-130656) was used. The membrane was washed with TBS-T (TBS containing 1% Tween-20), and then incubated (for 4 h) in either alkaline phosphatase (ALP) conjugated goat anti-rabbit (MERK, cat.# 62110080011730, 1:2000) or anti-mouse (MERK cat.# 621100480011730, 1:2000) antibody to detect the membrane bounded antibodies. Subsequently, ALP activity was detected with the substrates 5 bromo-4-chloro-3-indolyl phosphate di-sodium salt (BCIP) and nitro blue tetrazolium chloride (NBT) (cat.# S 3771; Promega Biotech Ltd., Madison, WI, USA), following the manufacturer’s instruction. Individuals’ blot images were obtained using Molecular Imager and each band trace quantity was measured (Chemi Doc XRS system, Image Lab 2 software (2.0) Bio-Rad laboratories Inc., Hercules, CA, USA). Differences from the control group were presented in relative fold [36,45,46,47]. All uncropped Western blot images are provided in Supplementary Materials.

### 2.11. Statistical Analysis

KyPlot (ver 1.0) was used to plot the values (mean ± standard error of the mean (SEM)) as a graphical representation. The significant difference among the experimental groups (CON, PNS, PNS + BME) were tested with one-way analysis of variance (ANOVA) followed by post hoc (Bonferroni test) analysis (Sigma Stat version 3.1). Significant difference among groups (* *p* < 0.05, ** *p* < 0.01, *** *p* < 0.001) and NS, not significantly different were indicated wherever required.

## 3. Results

### 3.1. Behavioral Results

#### 3.1.1. Elevated Plus Maze

**Figure 1:**
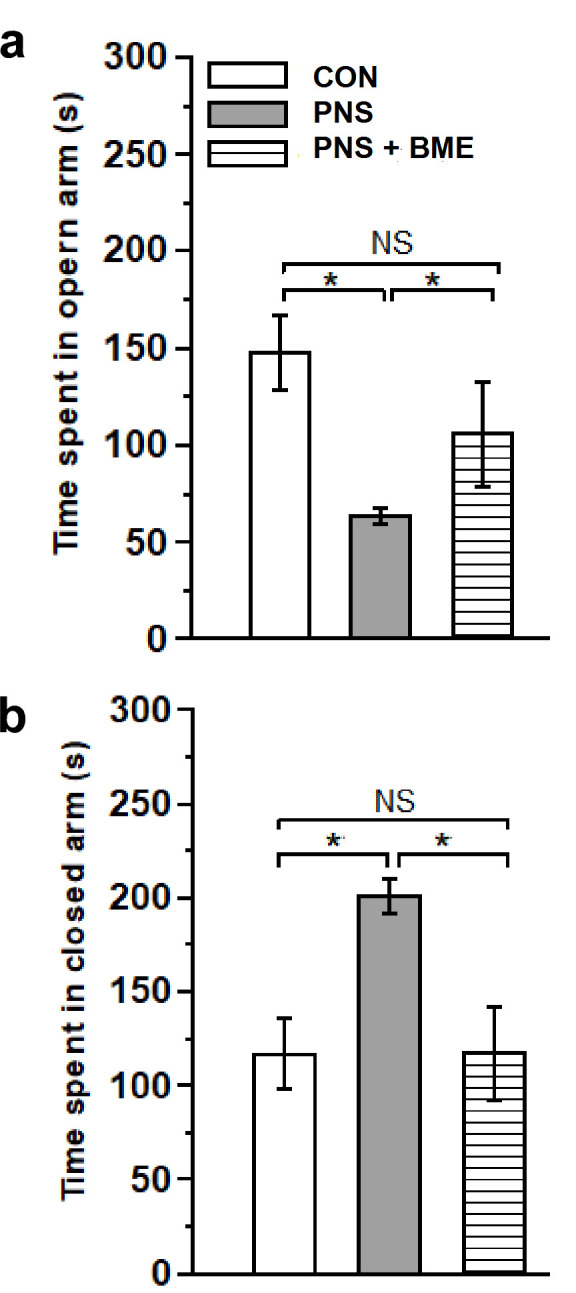
Exposure to BME treatment and resilience effect on the prenatal stress (PNS)-induced anxiety-like behaviors in the offspring. Behavioral profile in the elevated plus maze (EPM). (**a**) Total time spent in the closed arm; (**b**) Total time spent in the open arm. The anxiety induced by the gestational stress was significantly reduced by the maternal exposure to the BME treatment. Data are expressed as mean ± SEM (control *n* = 7, PNS *n* = 6, PNS + BME *n* = 6). One-way ANOVA followed by Bonferroni post hoc test (for all pairwise multiple comparisons, statistical significance is indicated by * *p* < 0.05; NS—Not significant).

**Figure 2:**
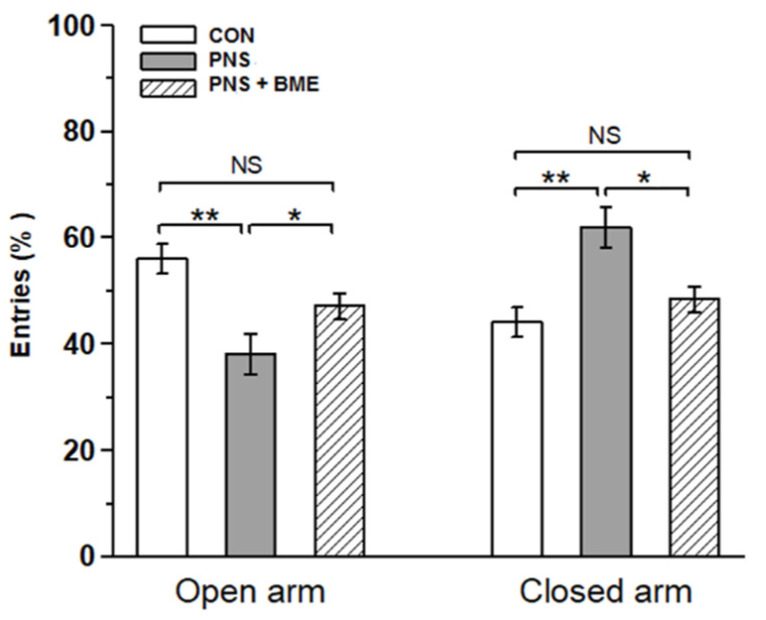
Effect of exposure to BME treatment on PNS-induced changes in offspring. Behavioral profile of EPM showed as the percentage of entries in the open arm and closed arm, maternal exposure to BME treatment and resilience effect on the anxiety-like behavior in the offspring. Data are expressed in percentage (mean ± SEM) calculated against the total number of entries (control *n* = 7, PNS *n* = 6, PNS + BME *n* = 6). One-way ANOVA followed by Bonferroni post hoc test (for all pairwise multiple comparisons, statistical significance is indicated by * *p* < 0.05 and ** *p* < 0.01; NS—Not significant).

Behavioral profile in EPM showed that exposure to the BME treatment reduced the PNS-induced anxiety-like behavior in their offspring. The observed behavioral data showed that there was a significant difference in the time spent in the open arm across the experimental groups (*F_(2,18)_* = 6.79, *p* < 0.01). Bonferroni post hoc comparative analysis suggests that, in the open arm, the PNS group spent less time than the control (*p* < 0.05) and the PNS + BME group (*p* < 0.05), but there was no significant difference between the control and the PNS + BME group (*p* = 1.00) (Figure 1a). In addition, for individual time spent in the closed arm, there was a significant difference across the study groups (*F_(2,18)_* = 7.300, *p* < 0.01). The post hoc analysis demonstrates that the PNS group spent more time than the control group (*p* < 0.05) and the PNS + BME group (*p* < 0.05). Whereas no difference was observed between the control group and the PNS + BME group (*p* = 0.984) (Figure 1b). Similarly, the PNS group resulted in a significant difference in total number of entries (*F_(2,37)_* = 3.76, *p* < 0.05). The post hoc analysis demonstrated that, unlike the control animals, the PNS group (*p* < 0.05) exhibited fewer entries and there was no significant difference between the PNS group and the PNS + BME group (*p* = 0.212) and the control group and the PNS + BME group (*p* = 1.00) (Figure 2). When the percentage of open entries was calculated, a significant difference was noted across the experimental group (*F_(2,18)_* = 9.53, *p* < 0.01). The post hoc analysis revealed that the PNS group was significantly different from the control group (*p* < 0.05) and the PNS + BME group (*p* < 0.05), but the control group was not significantly different from the PNS + BME group (*p* = 0.759). Similarly, a significant difference was observed across the groups regarding closed arm entries (*F_(2,18)_* = 9.22; *p* < 0.01). Subsequently, the post hoc comparison showed a significant difference between the control group and the PNS group (*p* < 0.01) and the PNS group and the PNS+ BME group (*p* < 0.05), where a significant difference was not observed between the control group and the PNS + BME group (*p* = 1.00).

#### 3.1.2. Spatial Memory

**Figure 3:**
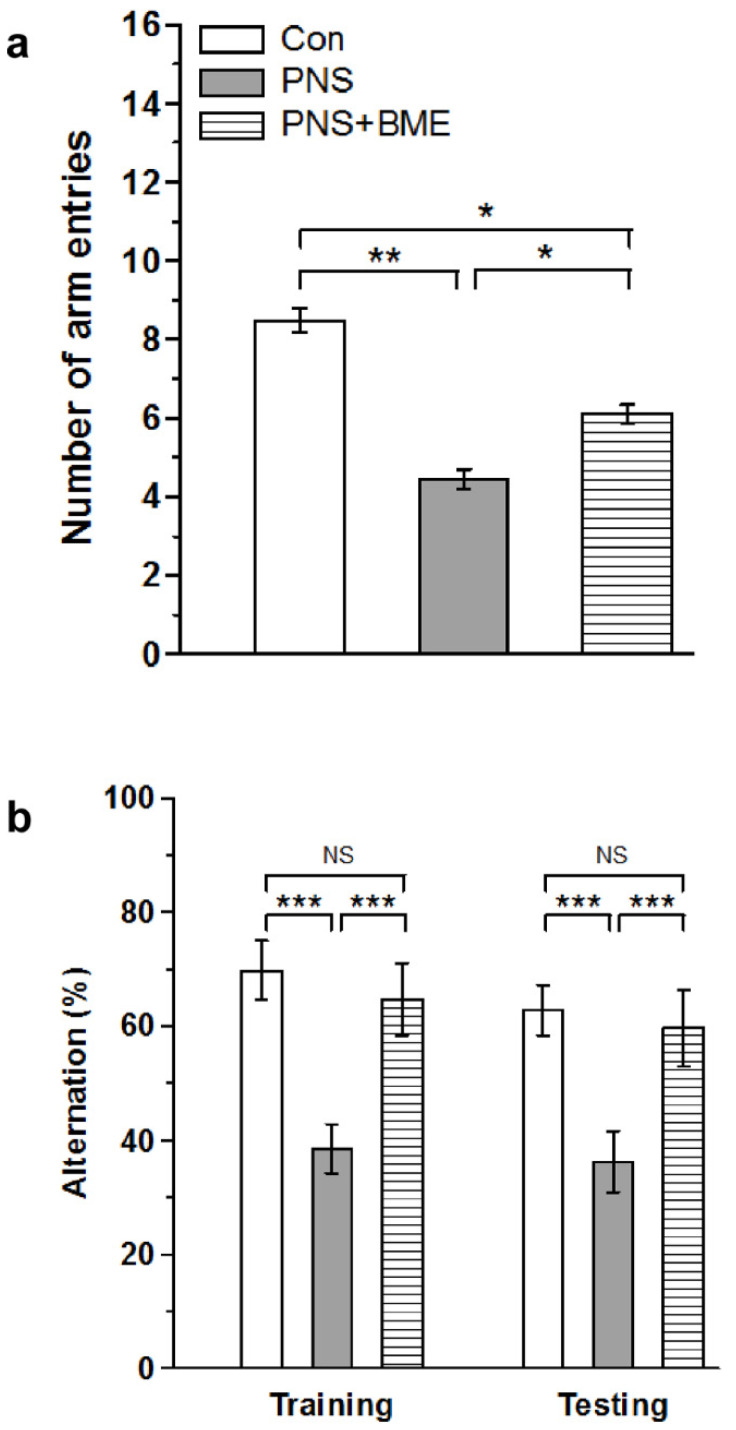
Exposure to BME treatment protects against PNS-induced memory impairment in offspring. The behavioral profile in the Y-maze. (**a**) The BME exposed group (**a**) made more arm entries than the PNS group (**b**) showed a higher percentage of alternations. Data are expressed as mean ± SEM (control *n* = 7, PNS *n* = 6, PNS + BME *n* = 6). One-way ANOVA followed by Bonferroni post hoc test (for all pairwise multiple comparisons, statistical significance is indicated by * *p* < 0.05, ** *p* < 0.01 and *** *p* < 0.001; NS—Not significant).

In addition, the Y-maze analysis showed a significant effect of the variable group (*F_(2,37)_ =* 14.28, *p* < 0.001), and the post hoc comparison analysis indicated that, with reference to the control group, the PNS group spent significantly less arm entries (*p* < 0.001), as well as the PNS + BME group (*p* <0.01). However, the PNS group made significantly less entries than the PNS + BME group (*p* < 0.05) (Figure 3a). When we estimated the percentage of alternation during training, we found a significant difference across the groups (*F_(2,37)_ =* 17.42, *p* < 0.001). The post hoc analysis revealed that the PNS group exhibited a lower percentage of alternation than the control group (*p* < 0.001) and the PNS + BME group (*p* < 0.001), but a significant difference was not observed between the control group and the PNS + BME group (*p* = 0.421). Similarly, during testing, the recorded alternation between groups was significantly different (*F_(2,37)_ =* 21.468, *p* < 0.001). The post hoc analysis showed that the PNS individuals displayed a lower percentage of alternation than the control group (*p* < 0.001) and the PNS + BME group (*p* < 0.001). In fact, we did not find a significant difference between the control and the PNS + BME groups *(p* = 0.11) (Figure 3b). The observed behavioral data in the EPM and the Y-maze tests demonstrated that the exposure to the BME treatment protected against prenatal stress-induced anxiety-like behavior and memory impairment in their offspring.

### 3.2. Hormonal Results

#### 3.2.1. Effect of Exposure to BME Treatment on Prenatal Stress-Induced Changes in Corticosterone and Adrenocorticotropic Hormone

**Figure 4:**
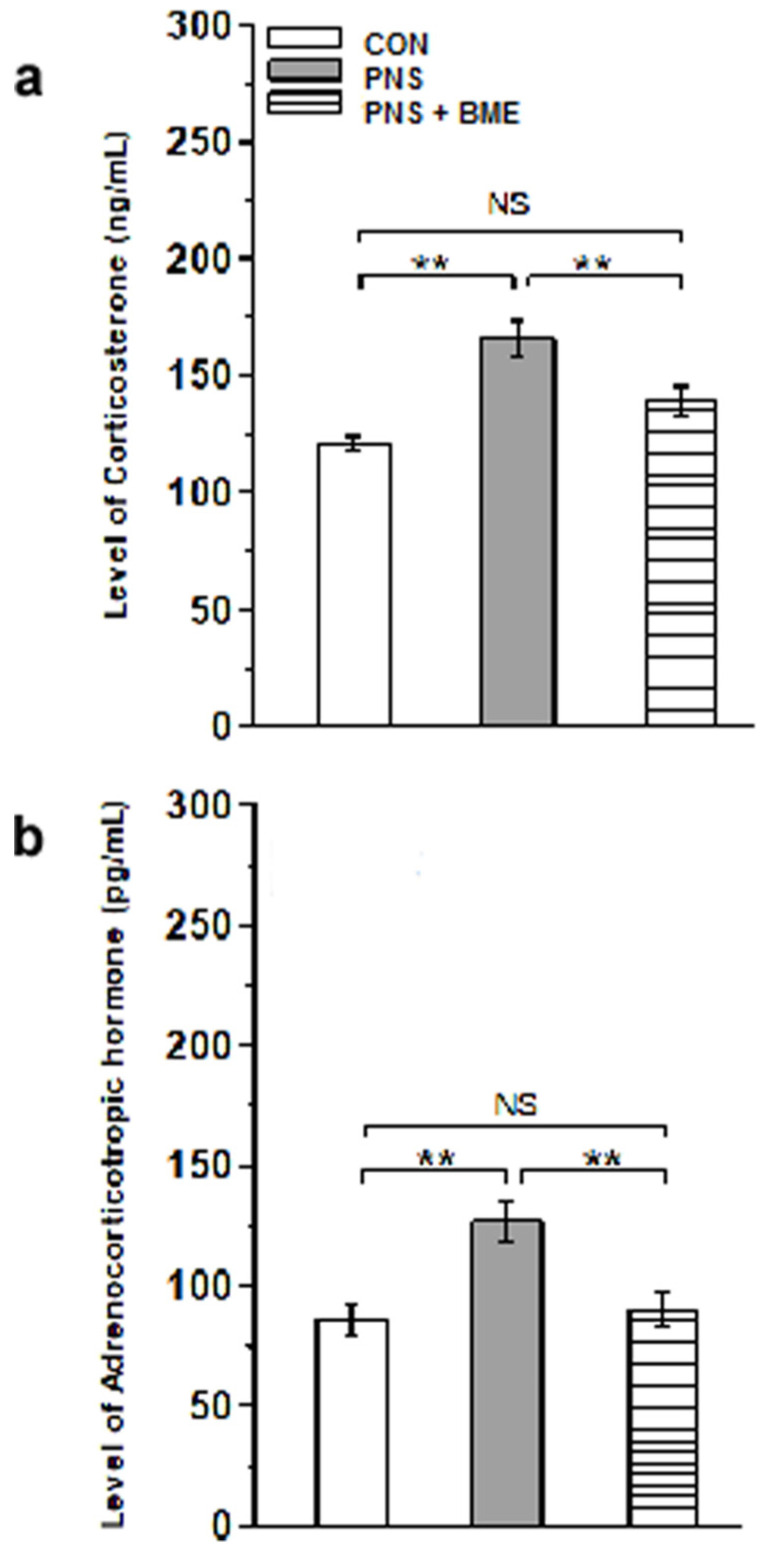
Maternal exposure to BME treatment and the resilience effect on the PNS-induced changes in offspring plasma. (**a**) Corticosterone (CORT); (**b**) Adrenocorticotropic hormone (ACTH) level. Exposure to BME treatment significantly reduced the PNS-induced CORT and ACTH level. Data are expressed as mean ± SEM (*n* = 6 for each group). One-way ANOVA followed by Bonferroni post hoc test (for all pairwise multiple comparisons, statistical significance is indicated by ** *p* < 0.01; NS—Not significant).

Subsequently, we estimated the level of corticosterone and adrenocorticotropic hormone in the plasma of the experimental groups. As shown in Figure 4a, a significant effect was demonstrated across the groups (*F_(2,17)_* = 104.78; *p* < 0.001).The post hoc test revealed significant changes in the level of corticosterone of the PNS group as compared with the control (*p <* 0.01) and PNS+ BME groups (*p <* 0.01). However, exposure to BME treatment significantly reduced the PNS-induced elevation in corticosterone of the PNS group, and therefore no significant difference between the control and PNS + BME groups (*p =* 0.078). Similarly, we found a significant increase in the level of adrenocorticotropic hormone of the PNS group as compared with the level of the control group (*F_(2,37)_* = 64.16; *p* < 0.001). The post hoc analysis indicated a significant increase in the adrenocorticotropic hormone level of the PNS group as compared with the control group (*p* < 0.01) and the PNS + BME group (*p* < 0.01). However, there was no significant difference between the PNS + BME and control groups (*p* = 0.844) (Figure 4b). The above results indicate that PNS increases the level of corticosterone and adrenocorticotropic hormone but exposure to BME treatment had resilience effect on the PNS-induced CORT and ACTH level, which could correlate with the observed anxiolytic behavior in the BME supplemented group.

#### 3.2.2. Effect of Exposure to BME Treatment on Prenatal Stress-Induced Changes on Glucocorticoid Receptor

**Figure 5:**
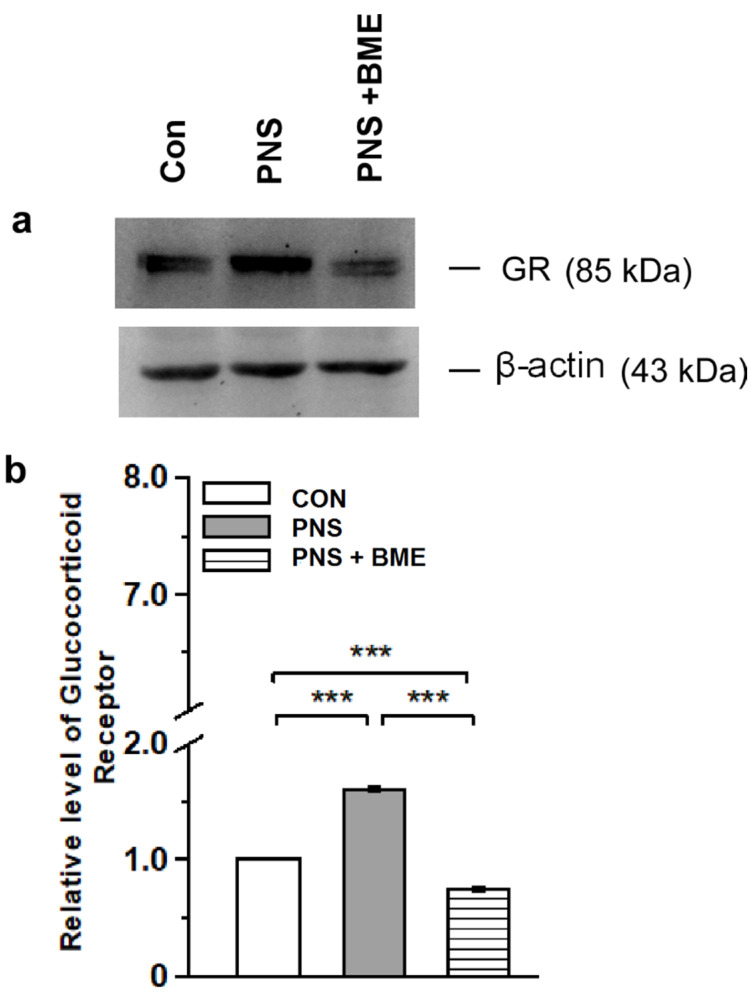
Effect of exposure to BME treatment on PNS-induced changes in offspring’s glucocorticoid receptor (GR) level in amygdale. (**a**) Representative Western blot showing that the level of GR varied across the experimental group; (**b**) Estimated level of GR suggests that exposure to BME treatment has a resilience effect on the PNS-induced changes. Data are expressed as mean ± SEM (*n* = 6/each group). One-way ANOVA followed by Bonferroni post hoc test (for all pairwise multiple comparisons, statistical significance is indicated by *** *p* < 0.001).

As shown in Figure 5 (Supplementary Figure S3), the level of glucocorticoid receptor expression was significantly different across the groups (*F_(2,17)_* = 278.96, *p* < 0.001). The post hoc comparison suggested that level of glucocorticoid receptor was significantly higher in the PNS group as compared with the control group (*p* < 0.001) and the PNS + BME group (*p* < 0.001). However, the level of glucocorticoid receptor was significantly lower in the PNS group exposed to BME treatment as compared with the control group (*p* < 0.001). The observed results suggest that PNS alters glucocorticoid receptor expression but exposure to BME treatment has a possible resilience effect on the PNS-induced changes in glucocorticoid receptor.

### 3.3. Western Blot Results

#### 3.3.1. Effect of Exposure to BME Treatment on Prenatal Stress-Induced Neuronal Apoptosis

**Figure 6:**
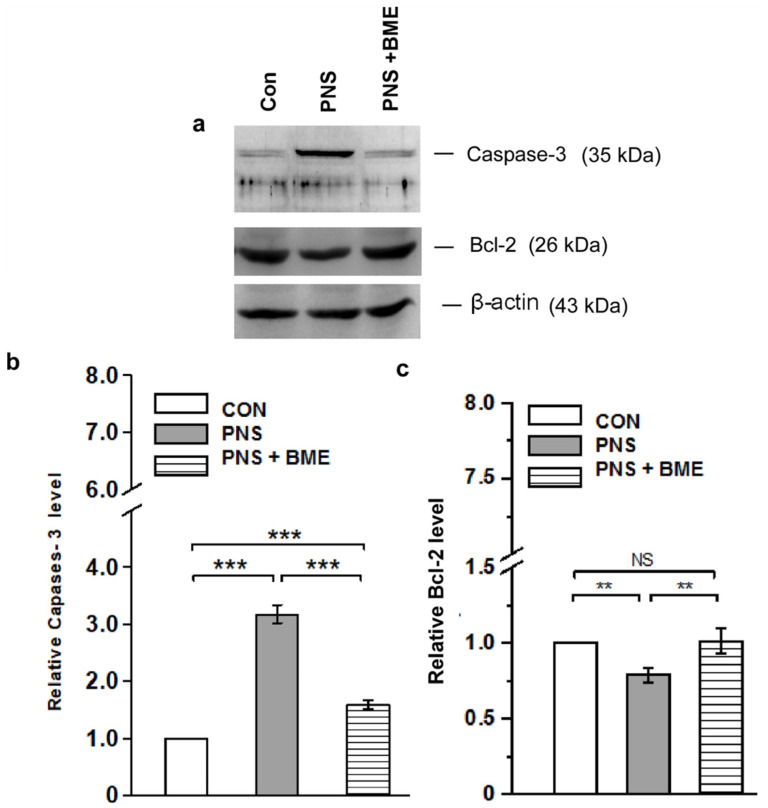
Effect of exposure to BME treatment on PNS-induced changes on caspase-3 and Bcl-2 level in amygdale. (**a**) Representative Western blot showing that the levels of caspase-3 and Bcl-2 varied across the experimental groups. Quantitative estimation of caspase-3 (**b**) and Bcl-2 (**c**) showing that exposure to BME treatment induces an opposite pattern of expression. Data are expressed as mean ± SEM (*n* = 6/group). One-way ANOVA followed by Bonferroni post hoc test (for all pairwise multiple comparisons, statistical significance is indicated by ** *p* < 0.01 and *** *p* < 0.001; NS—Not significant).

Furthermore, we examined the level of caspase-3 and Bcl-2 to measure the PNS-induced neuronal damage and the effect of exposure to BME treatment. The Western blot analysis showed that PNS induced the expression of caspase-3 (Figure 6a, Supplementary Figure S4). The analysis revealed that the level of caspase-3 significantly varied across the groups (*F_(2,17)_ =* 1714.3, *p* < 0.001). The post hoc analysis demonstrated that level of caspase-3 was significantly higher in the PNS group as compared with the control (*p* < 0.001) and the PNS + BME groups (*p* < 0.001). Exposure to the BME treatment reduced the caspase-3 level, however, the level was significantly higher than in the control group (*p* < 0.001) (Figure 6b). Furthermore, the level of Bcl-2 varied significantly across the experimental groups (*F_(2,17)_ =* 74.2, *p* < 0.001). The post hoc analysis indicated that the level of Bcl-2 was significantly lower in the PNS group than in the control (*p* < 0.01) and PNS + BME groups (*p* < 0.01). This showed that exposure to BME treatment elevated the Bcl-2 level, however, a significant difference was not detected between the control and the PNS+ BME groups (*p* = 0.092) (Figure 6c). Taken together, the observed results suggest that exposure to BME treatment protects against PNS-induced neuronal damage by balancing the expression of caspase-3 and Bcl-2.

#### 3.3.2. Effect of Exposure to BME Treatment on Prenatal Stress-Induced Changes in Synaptophysin and Synaptotagmin-1

**Figure 7:**
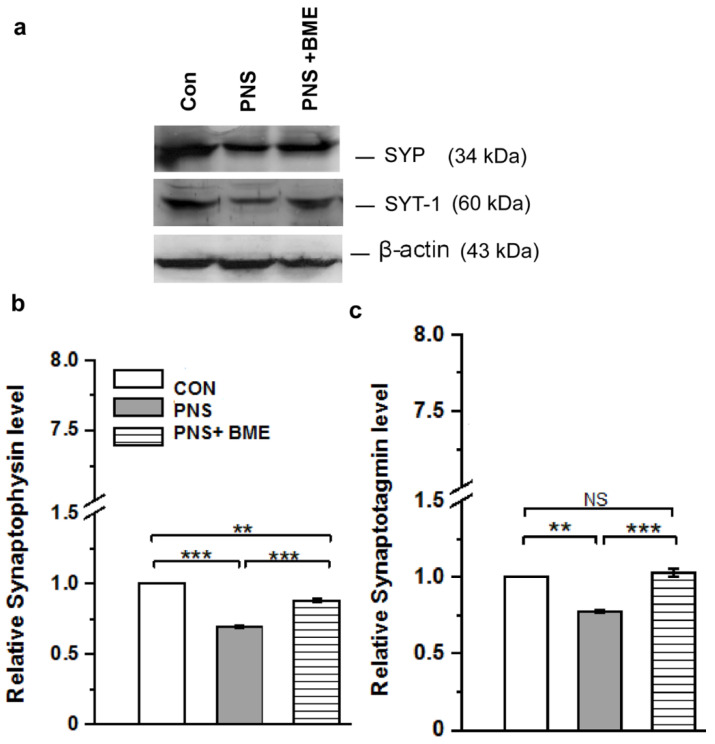
Exposure to BME treatment protects against PNS-induced changes in synaptic proteins. (**a**) Representative Western blots showing that the level synaptophysin (SYP) and synaptotagmin-1 (SYT-1) in the experimental groups; (**b**) SYP and (**c**) SYT-1 were significantly increased in the BME supplemented group. Data are expressed as mean ± SEM (*n* = 6/each group). One-way ANOVA followed by Bonferroni post hoc test (for all pairwise multiple comparisons, statistical significance is indicated by ** *p* < 0.01 and *** *p* < 0.001; NS—Not significant).

Furthermore, we found that exposure to BME treatment had a resilience effect on the PNS-induced changes in synaptic proteins (Figure 7a, Supplementary Figure S5). In addition, the level of synaptophysin was also altered by PNS. The observed changes in the level of synaptophysin was significantly different across the groups (*F_(2,17)_* = 2097.45, *p* < 0.001). The post hoc comparison showed that synaptophysin was significantly higher in the PNS group than that of the control group (*p* < 0.001) but significantly lower than that of the PNS + BME group (*p* < 0.001), whereas the level of synaptophysin was significantly less than the control group (*p* < 0.001) (Figure 7b). One-way ANOVA showed a significant difference across the experimental groups (*F_(2,17)_ =* 528.20, *p* < 0.001), and the post hoc comparison showed that the level of synaptotagmin-1 was significantly lower in the PNS group than the control (*p* < 0.01) and PNS+ BME group (*p* < 0.001). However, the detected synaptotagmin-1 level in the control group was not significantly different from individuals exposed to BME treatment (*p* = 0.08) (Figure 7c).

#### 3.3.3. Effect of Exposure to BME Treatment on Prenatal Stress-Induced Changes on 5-HT_1A_ and 5-HT_2C_ Receptors

**Figure 8:**
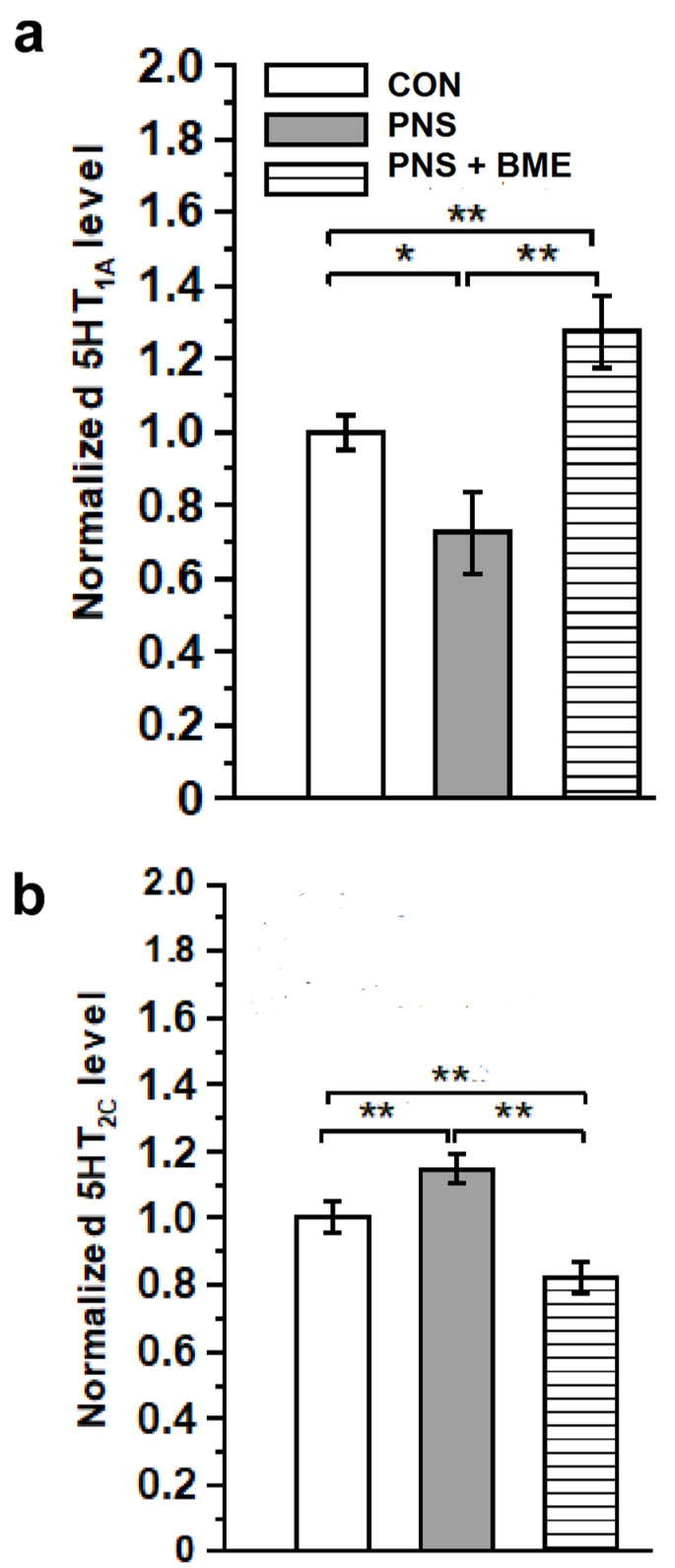
Exposure to BME treatment protects the PNS-induced alternations in the serotonergic system. Quantitative real-time PCR analysis showing that exposure to BME treatment significantly ameliorates the PNS-induced changes in (**a**) 5-HT_1A_ and (**b**) 5-HT_2C_ receptor expression. Data are expressed as mean ± SEM (*n* = 6/group). One-way ANOVA followed by Bonferroni post hoc test (for all pairwise multiple comparisons, statistical significance is indicated by * *p* < 0.05 and ** *p* < 0.01).

Subsequently, we estimated the expression level of 5-HT_1A_ and 5-HT_2C_ receptors in the experimental groups (Supplementary Figure S6). The one-way ANOVA analysis showed a significant difference in the estimated level of 5-HT_1A_ between the groups (*F_(2,17)_* = 44.94, *p*< 0.001). The post hoc analysis indicated that 5-HT_1A_ receptor expression was significantly lower in the PNS group than in the control (*p* < 0.05) and PNS + BME groups (*p* < 0.01). In fact, the level of 5-HT_1A_ was significantly higher in the PNS + BME group than in the control group (*p* <0.01) (Figure 8a). When we examined the 5-HT_2C_ receptor, the level of expression between groups varied significantly (*F_(2,17)_ =* 390.92, *p*< 0.001). The post hoc comparison revealed that the level of 5-HT_2C_ receptor expression was elevated in the PNS group as compared with the control (*p* < 0.01) and PNS + BME groups (*p* < 0.01). Exposure to the BME treatment significantly reduced the PNS-induced changes in 5-HT_2C_ receptor, and therefore a significant difference was observed between the control and PNS + BME groups (*p* < 0.001) (Figure 8b). The obtained data demonstrates that exposure to BME treatment differentially regulates 5-HT1A and 5-HT_2C_ receptors, with a possible resilience effect on the PNS-induced impact on the serotonergic system.

#### 3.3.4. Effect of Exposure to BME Treatment on Gestational Stress-Induced Changes on Neurogranin (Ng) and CaMKII Signaling

**Figure 9:**
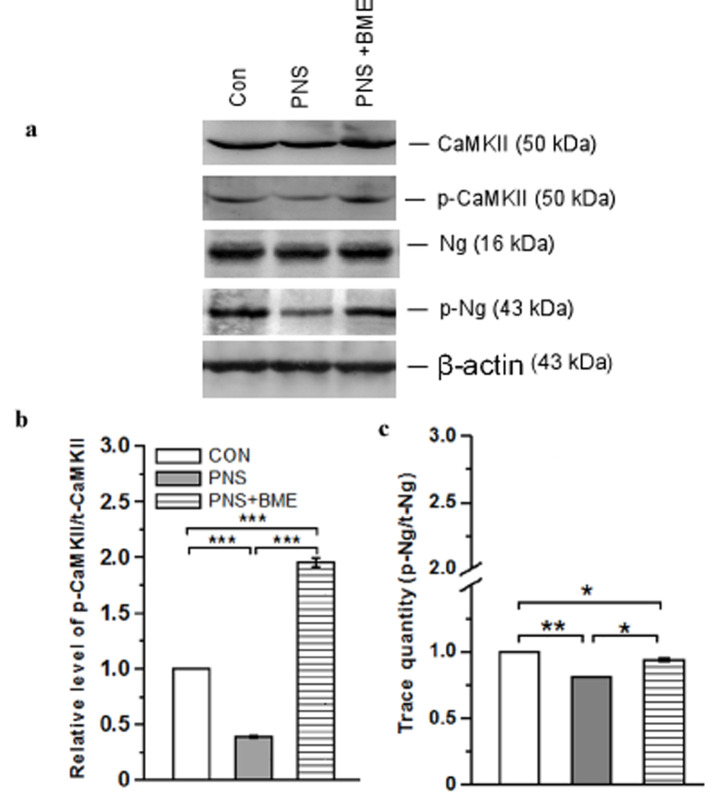
Exposure to BME treatment protects against PNS-induced changes in the activation of CaMKII. (**a**) Representative Western blot showing variations in the level of CaMKII/p-CaMKII and Ng/p-Ng. Estimated ratio of phosphorylation of CaMKII (**b**) and Ng/p-Ng (**c**) in experimental groups. Data are expressed as mean ± SEM (*n* = 6/each group). One-way ANOVA followed by Bonferroni post hoc test (for all pairwise multiple comparisons, statistical significance is indicated by * *p* < 0.05, ** *p* < 0.01 and *** *p* < 0.001).

Subsequently, we examined the effect of PNS on activation of CaMKII. The western blot analysis demonstrated that the level of CaMKII was altered (Figure 9a, Supplementary Figure S7). The one-way ANOVA reported a significant difference across the variable groups (*F_(2,17)_* = 132.10, *p* < 0.001). The post hoc comparison analysis showed that the level of p-CaMKII/CaMKII was significantly reduced in the PNS group (*p* < 0.001) as compared with the control and PNS + BME groups (*p* < 0.001). Furthermore, the level of p-CaMKII/CaMKII was significantly higher in the PNS + BME group than in the control group (*p* < 0.001) (Figure 9b).

Next, we examined whether the neurogranin expression was associated with the CaMKII expression. Our analysis revealed that the level of neurogranin expression among the groups was significantly different (*F_(2,17)_* = 94.08, *p* < 0.001). In addition, the post hoc analysis showed that the level of p-Ng/Ng in the PNS group was significantly lower than in the control (*p* < 0.01) and PNS + BME groups (*p* < 0.05). The comparison analysis showed that the level of p-Ng/Ng was significantly lower in the PNS + BME group than in the control group (*p* < 0.05) (Figure 9c). These analyses suggest that PNS reduced the level of phosphorylation of Ng and CaMKII but the exposure to BME treatment reduced the effect.

#### 3.3.5. Effect of Exposure to BME Treatment on Gestational Stress-Induced Changes on *N*-Methyl-D-Aspartate Receptors (2A,2B)

**Figure 10:**
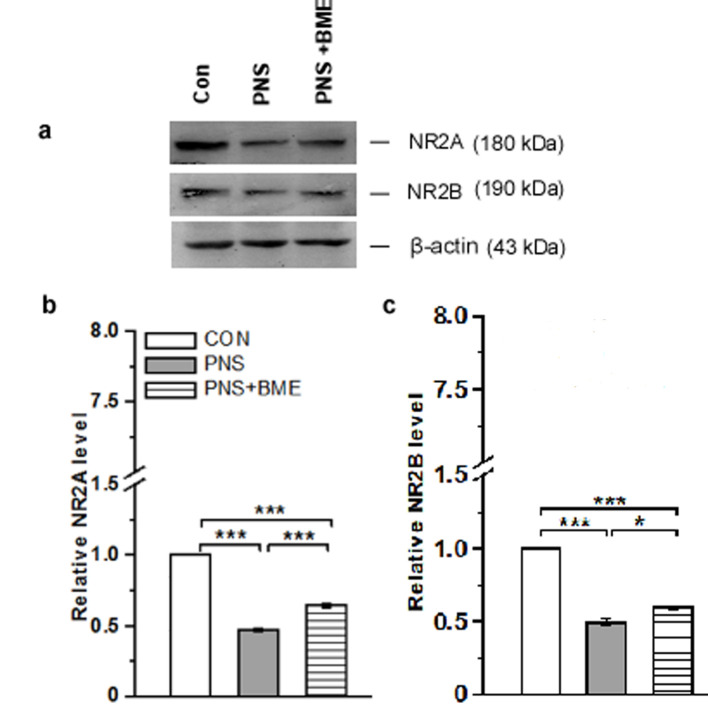
Exposure to BME treatment protects against the PNS-induced changes in NR2A and NR2B. (**a**) Representative Western blots showing that the level of NR2A and NR2B; (**b**) NR2A and (**c**) NR2B levels were significantly altered by exposure to BME treatment. Data are expressed as mean ± SEM (*n* = 6/each group). One-way ANOVA followed by Bonferroni post hoc test (for all pairwise multiple comparisons, statistical significance is indicated by * *p* < 0.05 and *** *p* < 0.001).

Exposure to BME treatment protects against the PNS-induced alteration in *N*-methyl-D-aspartate receptors (Figure 10a, Supplementary Figure S8). The Western blot analysis exhibited the differential expression pattern in the *N*-methyl-D-aspartate receptors among the experimental groups. One-way ANOVA showed that the level of NR2A varied significantly across the experimental groups (*F_(2,17)_* = 719.59, *p* < 0.001), and the post hoc analysis suggested that the level of NR2A was significantly low in PNS group than in the control (*p* < 0.001) and PNS + BME groups (*p* < 0.001). BME treatment protects against the PNS-induced changes in NR2A expression, however, the level of NR2A was significantly low in the PNS + BME group (*p* < 0.001) (Figure 10b). Similarly, the NR2B level was also altered and the variations among the groups were significantly different (*F_(2,17)_* = 72.00, *p* < 0.01). The post hoc analysis revealed that the NR2B level was significantly lower in the PNS group than in the control (*p* < 0.001) and PNS + BME groups (*p* < 0.05). Exposure to BME treatment protected against the PNS-induced effect, however, the NR2B levels in the control and PNS + BME groups were significantly difference (*p* < 0.001) (Figure 10c). The observed results suggest that exposure to BME treatment had a resilience effect on the PNS-induced changes in *N*-methyl-D-aspartate receptors.

#### 3.3.6. Effect of Exposure to BME Treatment on Postnatal Stress-Induced Changes in Postsynaptic Density Protein-95

**Figure 11:**
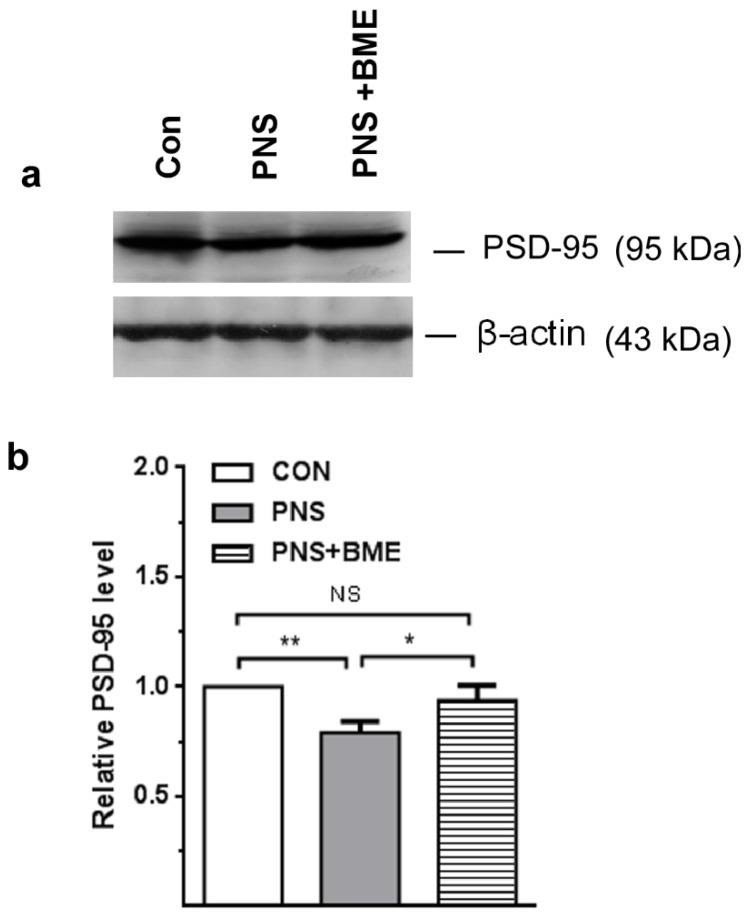
Effect of exposure to BME treatment on postsynaptic density protein 95 (PSD-95) in PNS offspring. (**a**) Representative Western blot depicting the level of PSD-95 in the experimental groups; (**b**) Exposure to BME treatment significantly increased the expression of PSD-95. Data are expressed as mean ± SEM (*n* = 6/each group). One-way ANOVA followed by Bonferroni post hoc test (for all pairwise multiple comparisons statistical significance is indicated by * *p* < 0.05 and ** *p* < 0.01; NS—Not significant).

Furthermore, our analysis showed PNS-induced changes in expression of postsynaptic density protein-95 (Figure 11; Supplementary Figure S9), which varied significantly across the experimental groups (*F_(2,17_*_)_ = 88.62, *p* < 0.001). The post hoc analysis indicated that the level of postsynaptic density protein-95 in the PNS group was significantly lower than in the control (*p* < 0.01), and PNS + BME groups (*p* < 0.05). Furthermore, exposure to BME treatment elevated the postsynaptic density protein-95 level, which was not significantly different from the control group (*p* = 0.14) (Figure 11a). Taken together, these results demonstrated that exposure to BME treatment elevated the expression of postsynaptic density protein 95.

#### 3.3.7. Effect of Exposure to BME Treatment on Prenatal Stress-Induced Changes in Expression of Pro and Mature Brain-Derived Neurotrophic Factor

**Figure 12:**
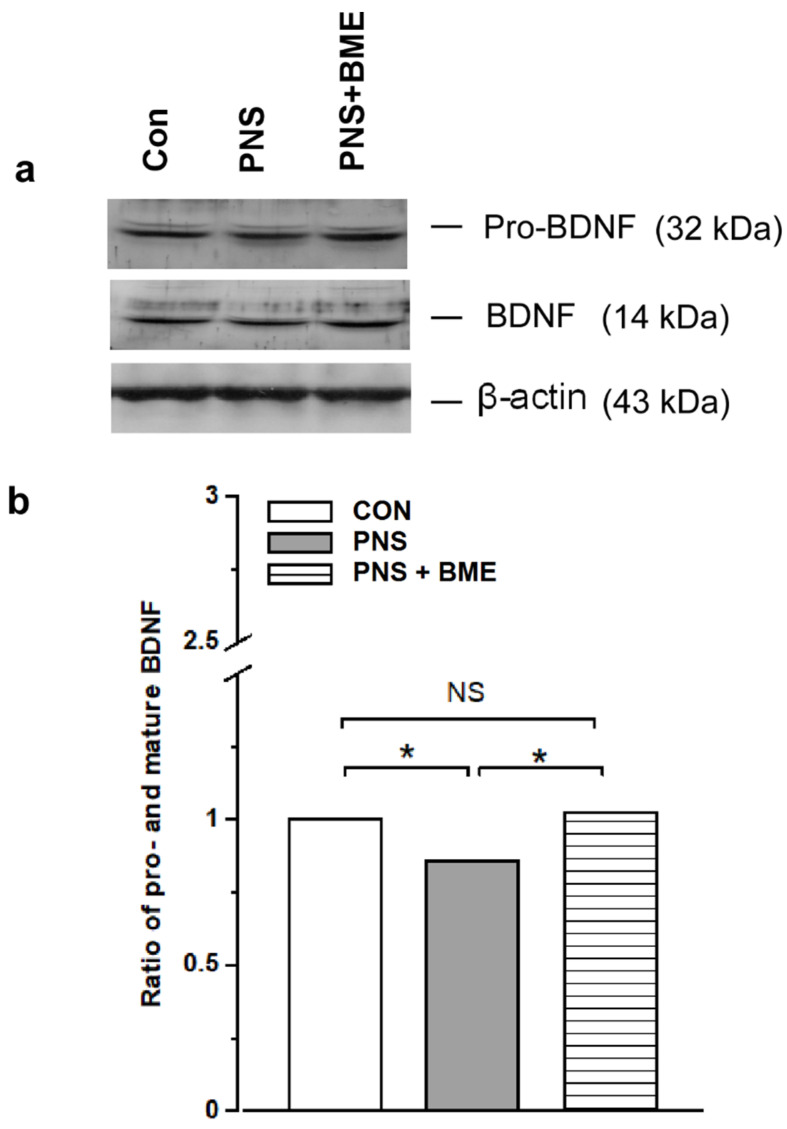
Effect of exposure to BME treatment on pro and mature brain-derived neurotrophic factor (BDNF) in PNS offspring. (**a**) Representative Western blots depicting the level of pro BDNF and mature BDNF; (**b**) Exposure to BME treatment silences the PNS-induced changes in the ratio of proand mature BDNF. Data are expressed as mean ± SEM (*n* = 6/each group). One-way ANOVA followed by Bonferroni post hoc test (for all pairwise multiple comparisons, statistical significance is indicated by * *p* < 0.05; NS—Not significant).

The level of pro and mature brain-derived neurotrophic factor was altered by PNS (Figure 12a; Supplementary Figure S10). One-way ANOVA found that ratio of pro and mature brain-derived neurotrophic factor varied significantly across the experimental groups (*F_(2,17)_* = 132.10, *p* < 0.001). The post hoc analysis demonstrated that the estimated ratio of pro and mature BDNF in the PNS group was significantly lower as compared with the control (*p* < 0.05) and PNS + BME groups (*p* < 0.05). Interestingly, a significant difference was not detected between the control and PNS + BME groups (*p* = 0.495) (Figure 12b). the observed results suggest that PNS alters the conversion of mature brain-derived neurotrophic factor but the exposure to BME treatment has a resilience effect on the PNS-induced effect on brain-derived neurotrophic factor maturation.

## 4. Discussion

Prenatal stress can alter the architecture of the developing brain program [51], more specifically the amygdala region [52] by a mother’s physiological and emotional environment [53]. The outcomes of animal and human studies have linked maternal stress and epigenetic changes; PNS has been known to alter the methylation pattern of GR, and thereby influence the HPA axis regulation in offspring and induce depression and anxiety-like behavior [54]. In this study, we observed that PNS induced anxiety-like behavior in offspring, which was similar to earlier reports in other models [55,56] and we validated our social defeat observation model. However, the observed anxiolytic behavior in the PNS + BME group showed that exposure to BME treatment had a resilience effect on prenatal stress and reduced the anxiety-like behavior in their offspring, as reported in other anxiety animal models [31,32]. Exposure to stress during gestation has been known to alter the level of stress hormone in the dam and also in the amniotic fluid [57], which can induce alterations in the HPA axis in offspring [58]. When the level of CORT and ACTH was estimated, we found that the level of CORT and ACTH was elevated in individuals experiencing PNS, as reported earlier [7]. However, exposure to BME treatment had a resilience effect on the PNS-induced changes and the level of CORT and ACTH was close to the basal level in their offspring. The adoptogenic properties of BME possibly have a resilience effect on the PNS-induced effect on CORT and ACTH [32,59]. We observed that the level of GR was up regulated in individuals who experienced PNS, and exposure to BME treatment resulted in down regulation of GR, which might be due to GR-CORT interactions [60,61]. In addition, it has been documented that stress induced an elevated level of CORT, overexpression of the GR known to activate oxidative mediators [62,63], and then up-regulation of the potential regulatory molecule caspase-3 in respond to the oxidative stress [63,64] and neuronal damage [65]. Caspase-3 is the key effector molecule in the caspase family, which is involved in the final execution of apoptosis, and activates the apoptosis process through the signal conduction pathway mediated by caspase-3 [66,67]. We observed that the level of caspase-3 was up regulated by PNS. Furthermore, we noted that exposure to BME treatment lowered the caspase-3 expression, suggesting that BME possibly reduced the oxidative stress, as reported earlier [32,68]. Interestingly, anti-apoptotic Bcl-2 was reduced by PNS, while exposure to BME treatment increased Bcl-2 expression, which has been known to suppress apoptosis [69,70]. Our data clearly demonstrate that the trends of caspase-3 and Bcl-2 expression are opposite. The levels of caspase-3 and Bcl-2 were dynamically balanced in this model. These results support the neuroprotective effects of BME in the PNS-exposed offspring and are consistent with those of another models [71,72].

The HPA axis receives serotonergic input, and its feedback mechanism it a critical regulator of behavior [73,74,75]. In line with earlier reports with other stress models [74,76,77], a reduced level of SYP was observed in the individuals who experienced PNS and displayed anxiety-like behavior. Whereas, exposure to BME treatment restored the level of SYP and behavior; the changes in level of SYP possibly associated with modulation of the HPA axis [75,77]. Notably, the HPA axis mediated changes in SYP have been known to drive the changes in SYT-1 [78,79]. Similarly, we found that PNS induced reduction of SYT-1 expression, however, exposure to BME treatment restored the level of SYT-1 and their behavior. Similar to other animal models, the observed lower level of SYT-1 was possibly associated with an imbalance in the synaptic vesicle exocytosis, which has been implicated in regulating synaptic transmission, synaptic strength, and plasticity [80,81]. Taken together, these results demonstrate that expression of SYP and SYT may be associated with synaptic plasticity and decreased long-term potentiation (LTP). Earlier studies have demonstrated that synaptic transmission could be mediated by Ca^2+^ dependent SYT-1 interaction with synaptic protein and 5-HT receptors [82,83]. In addition, activation/inhibition of 5-HT receptors regulates the CRH expression [84]. Stress-induced changes in synaptic transmission possibly induce adverse effects on serotonergic systems (level of 5-HT, 5-HT receptors, and SERT), affecting fetal development [85,86]. We found that the level of 5-HT_1A_ receptor expression was significantly decreased, whereas the 5-HT_2C_ receptor expression was elevated. In basolateral amygdale, the 5-HT_1A_ receptor was involving in neural circuits and its activation reduced fear-related anxiety [87]. PNS-induced reduction in the 5-HT_1A_ receptor could reflect the reactive anxiety-like behavior [88]. Exposure to BME treatment facilitates activation of the 5-HT_1A_ receptor and is possibly related to the observed anxiolytic behavior [18,20]. Earlier studies on stress model have demonstrated that up regulation of the 5-HT_2C_ receptor contributed to increased response to anxiety [21]. The underlying mechanism in the differential expression of the 5-HT receptor may be possibly associated with stress-mediated elevated level of corticosterone [89,90]. It is worthwhile mentioning that exposure to BME treatment and the resilience effect on the PNS-induced changes in 5-HT_1A_ and 5-HT_2C_ receptors were similar to the other stress models [91].

The CaMKII has been known to be more sensitive to 5-HT transmission, intracellular calcium level, and its kinase activity mediated by phosphorylation [20,21]. As reported by Fumagalli et al. [92], we observed that PNS did not alter the total CaMKII, but the level of phosphorylation was decreased. However, exposure to BME treatment elevated the phosphorylation level of CaMKII [93], which subsequently activated the target genes and may be linked with the observed anxiolytic behavior. Earlier studies have shown that at low concentrations or in the absence of Ca^2+^, Ng could bind with calmodulin (CaM), which was linked with the activity-dependent synaptic plasticity and LTP, a substrate for learning and memory [94,95]. In this study, we observed the level of Ng was significantly low in the PNS group, but BME treatment protected against the effect of PNS and the individuals showed less anxiety-like behavior and memory. Supporting our observation, earlier studies demonstrated that stress reduced the level of Ng and led to a reduction in LTP and a functional deficit in spatial learning [96,97]. The observed behavioral phenotype in this study could be linked with the PNS-induced reduction in Ng and CaMKII.

As reported earlier, PNS down regulates the NMDA receptor expression (NR2A, 2B) [98,99], possibly through the interaction of p-CaMKII [23]. It should be noted that exposure to BME treatment and the resilience effect on the PNS-induced effects on NR2A and NR2B receptor expression may be correlated with the observed anxiolytic behavior. Subsequently, we found that the PNS mediated effect on PSD-95 and further exposure to BME treatment increased the level of PSD-95, possibly due to the differential effects mediated expression [24]. Exposure to the stress has been known to alter the BDNF level either directly by glucocorticoid level or through the signaling molecules [7]. We found that the level of proand mature BDNF was decreased in the amygdala of offspring that experienced PNS, similar to another prenatal study report on hippocampus [100]. Stress reduced the proteolytic conversion of pro-BDNF to mature BDNF; therefore, the lower level of BDNF estimated in individuals who experienced PNS [27]. However, exposure to BME treatment increased the level of pro-BDNF, as well as the mature BDNF level. The observed mechanism in the present study could be the effect of BME treatment mediating BNDF level, as in earlier reports on other models [32,101].

## 5. Conclusions

In conclusion, behavioral data suggest that PNS induces anxiety-like behavior in offspring which may be linked to an alteration in the HPA axis (CORT, ACTH, GR) and overexpression of GR, activating oxidative stress and possibly the neuronal damage. Interestingly, exposure to BME treatment balances caspase-3 and Bcl-2 expression to protect the neurons. In addition, the feedback mechanism of the HPA axis and the serotonergic system alter synaptic proteins (synaptophysin, synaptotagmin-1), 5-HT receptors, phosphorylation of Ng and its interaction CaMKII, NMDA receptors (2A, 2B), PSD-95, and conversion of pro and mature BDNF. Exposure to BME treatment protects against the PNS-induced neuronal damage and changes in proand mature BDNF through the HPA axis and synaptic protein mediated signaling, which may be linked with the observed anxiolytic behavior in offspring.

## Supplementary Materials

The following are available online at https://www.mdpi.com/2076-3921/9/12/1229/s1. Supplementary Data-1: High performance liquid chromatography (HPLC) chromatogram of *Bacopa monnieri* extract, Supplementary Information: Supplementary figures showing all the uncropped full Western blot images. Supplementary Data for Western blot: Representative Western blots each band trace quantity.
