# Supplementary material for: Standardized Bacopa monnieri Extract Ameliorates Learning and Memory Impairments through Synaptic Protein, Neurogranin, Pro-and Mature BDNF Signaling, and HPA Axis in Prenatally Stressed Rat Offspring
Source: Antioxidants (Basel). 2020 Dec 4;9(12):1229. doi: 10.3390/antiox9121229 (PMC7761874; doi:10.3390/antiox9121229)
Supplement: Supplementary file 1 [file antioxidants-09-01229-s001.zip › Supplementary files/Supplementary data for western blot.pdf]

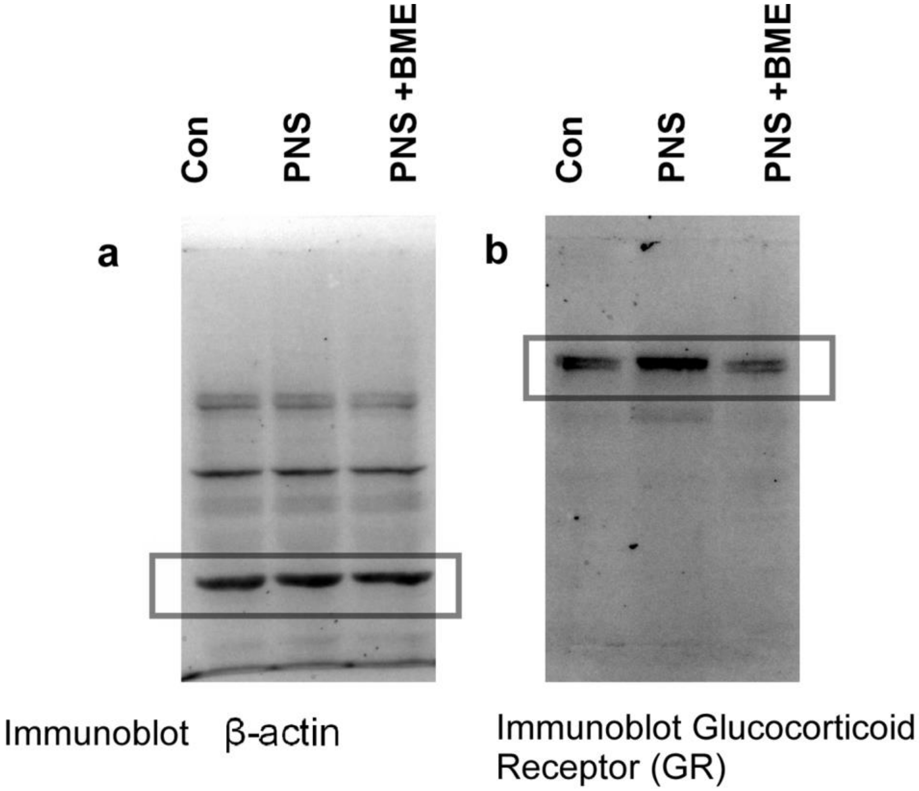

Supplementary Figure 3. Full immunoblot (uncropped) (a) β-actin (lower panel), (b) Glucocorticoid receptor (GR) (upper panel) used for figure 5 in the manuscript. Gray rectangles are the images cropped from each blot that are shown in the manuscript and each lane representing experimental groups (Con: Control; PNS: prenatal stress group; PNS+ BME: prenatal stress group treated with *B. monnieri* extract).

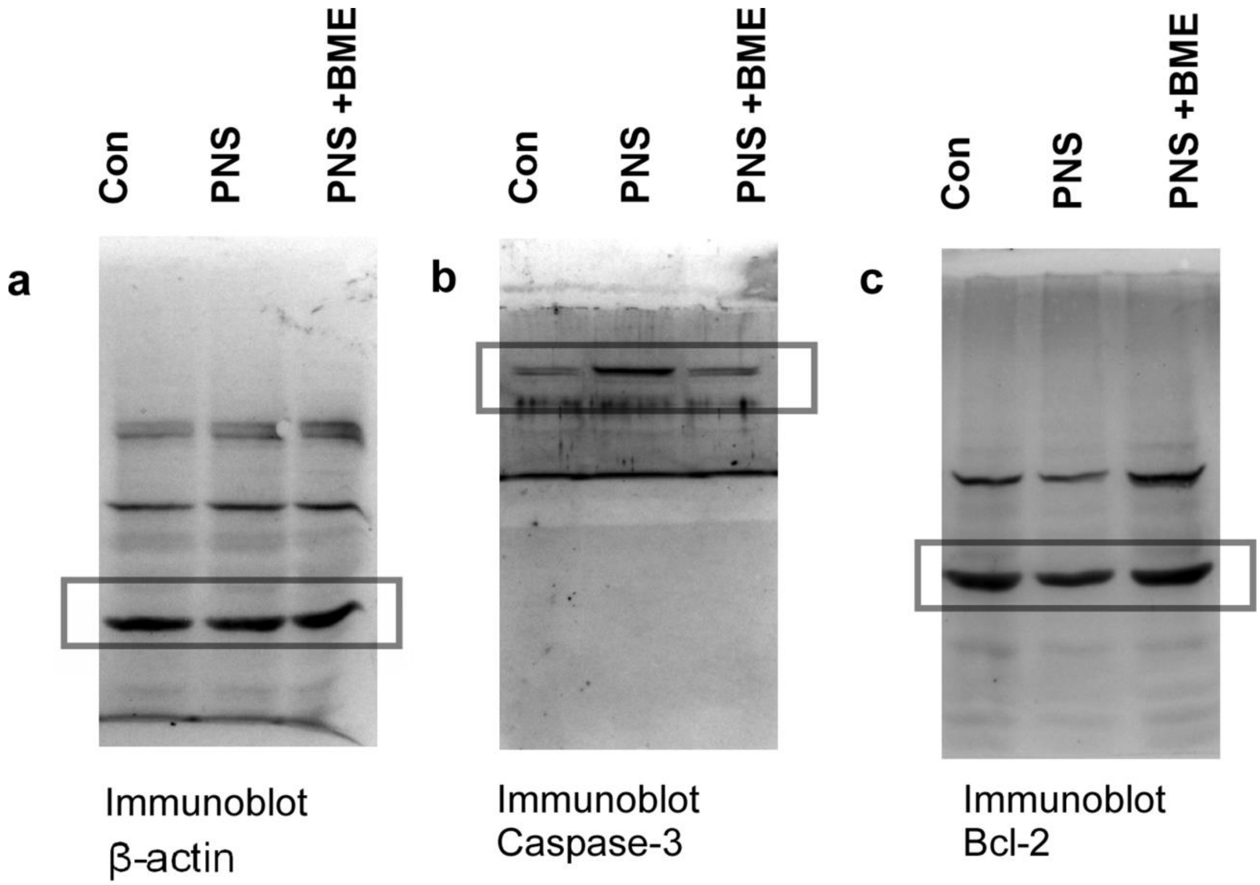

Supplementary Figure 4. Full immunoblot (uncropped) (a) β-actin (lower panel), (b) Caspase-3 (upper panel), (c) Bcl-2 (middle panel) used for figure 6 in the manuscript. Gray rectangles are the images cropped from each blot that are shown in the manuscript and each lane representing experimental groups (Con: Control; PNS: prenatal stress group; PNS+ BME: prenatal stress group treated with *B. monnieri* extract).

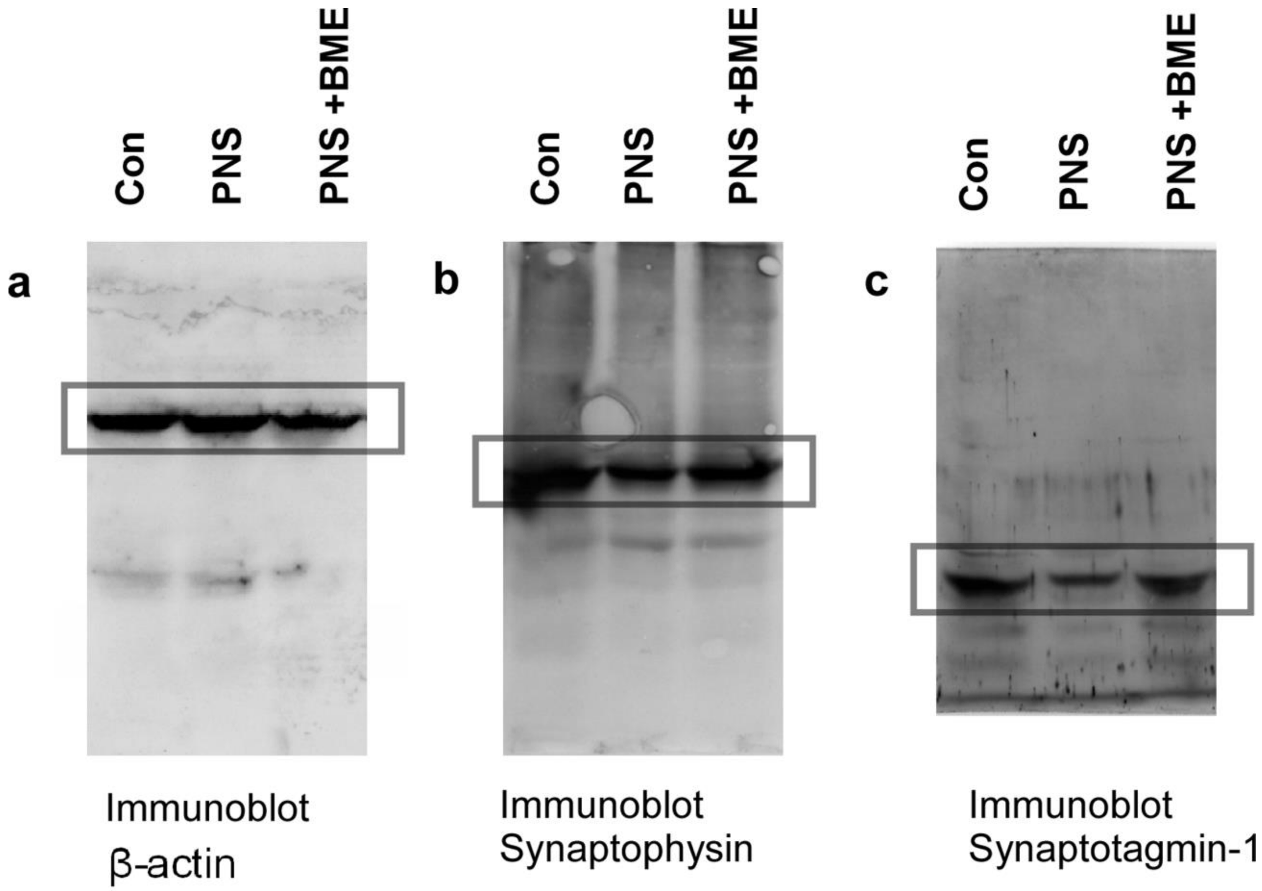

Supplementary Figure 5. Full immunoblot (uncropped) (a) β-actin (lower panel), (b) Synaptophysin (upper panel), (c) Synaptotagmine-1 (middle panel) used for figure 7 in the manuscript. Gray rectangles are the images cropped from each blot that are shown in the manuscript and each lane representing experimental groups (Con: Control; PNS: prenatal stress group; PNS+ BME: prenatal stress group treated with *B. monnieri* extract).

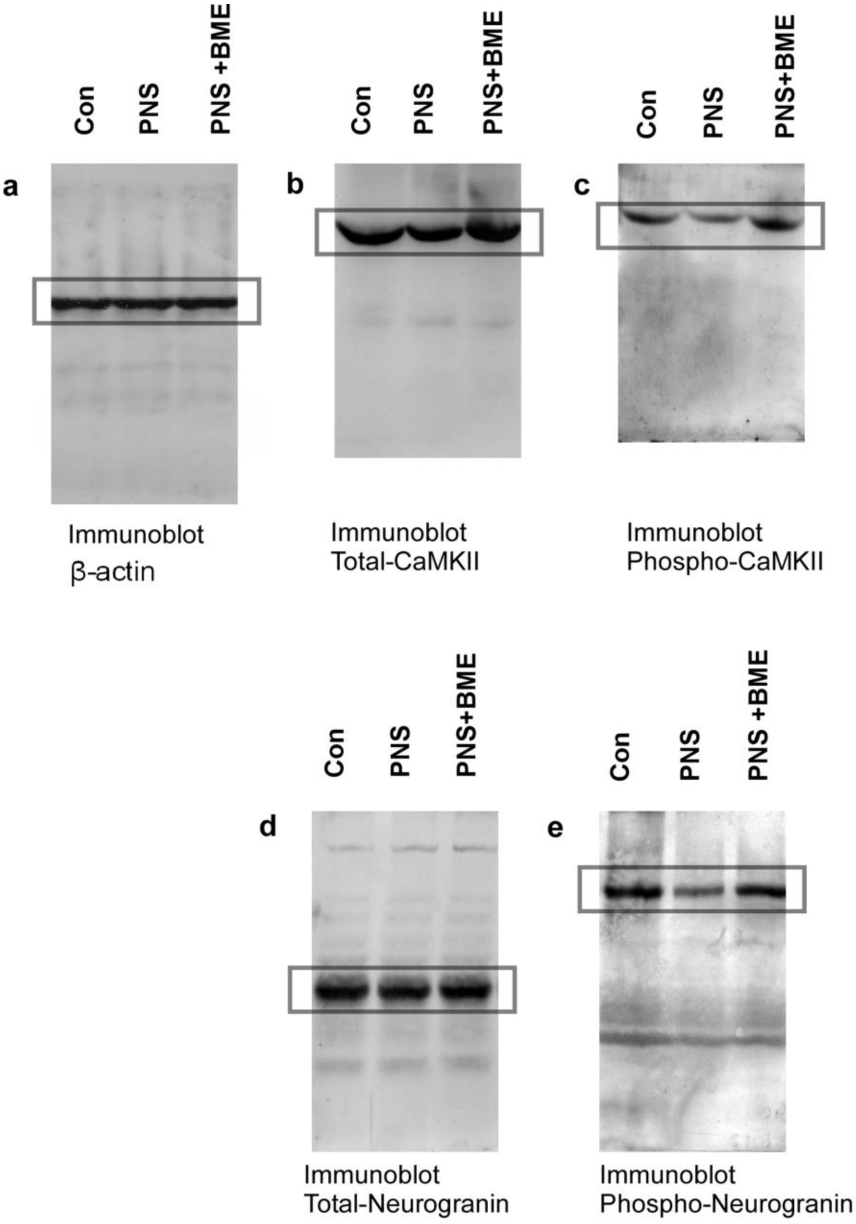

Supplementary Figure 7. Full immunoblot (uncropped) (a) β-actin (last panel), (b) total-CaMKII (first panel), (c) phosphorylated-CaMKII (second panel), (d) total-Neurogranin (third panel) and (e) phosphorylated-Neurogranin (fourth panel) used for figure 9 in the manuscript. Gray rectangles are the images cropped from each blot that are shown in the manuscript and each lane representing experimental groups (Con: Control; PNS: prenatal stress group; PNS+ BME: prenatal stress group treated with *B. monnieri* extract).

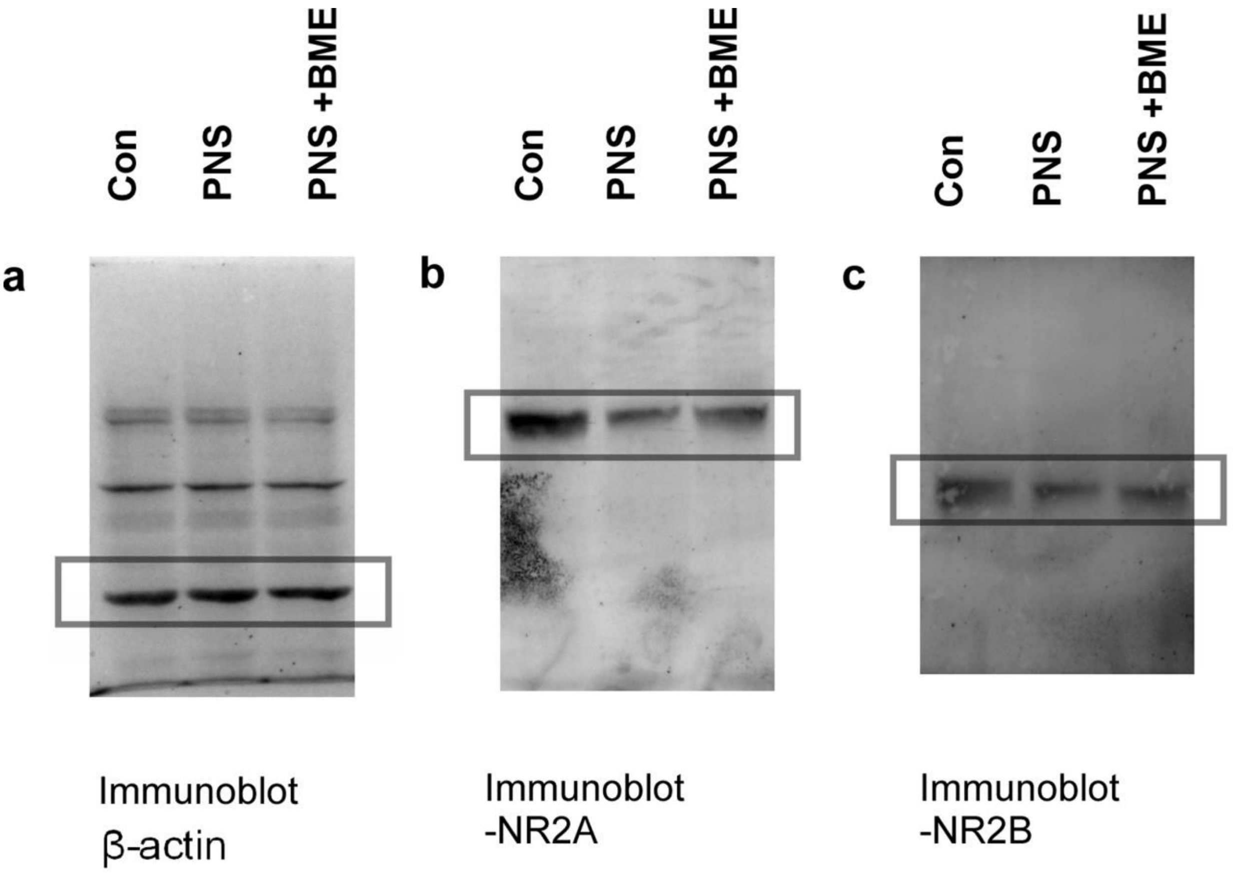

Supplementary Figure 8. Full immunoblot (uncropped) (a) β-actin (lower panel), (b) NR2A (upper panel), (c) NR2B (middle panel) used for figure 10 in the manuscript. Gray rectangles are the images cropped from each blot that are shown in the manuscript and each lane representing experimental groups (Con: Control; PNS: prenatal stress group; PNS+ BME: prenatal stress group treated with *B. monnieri* extract).

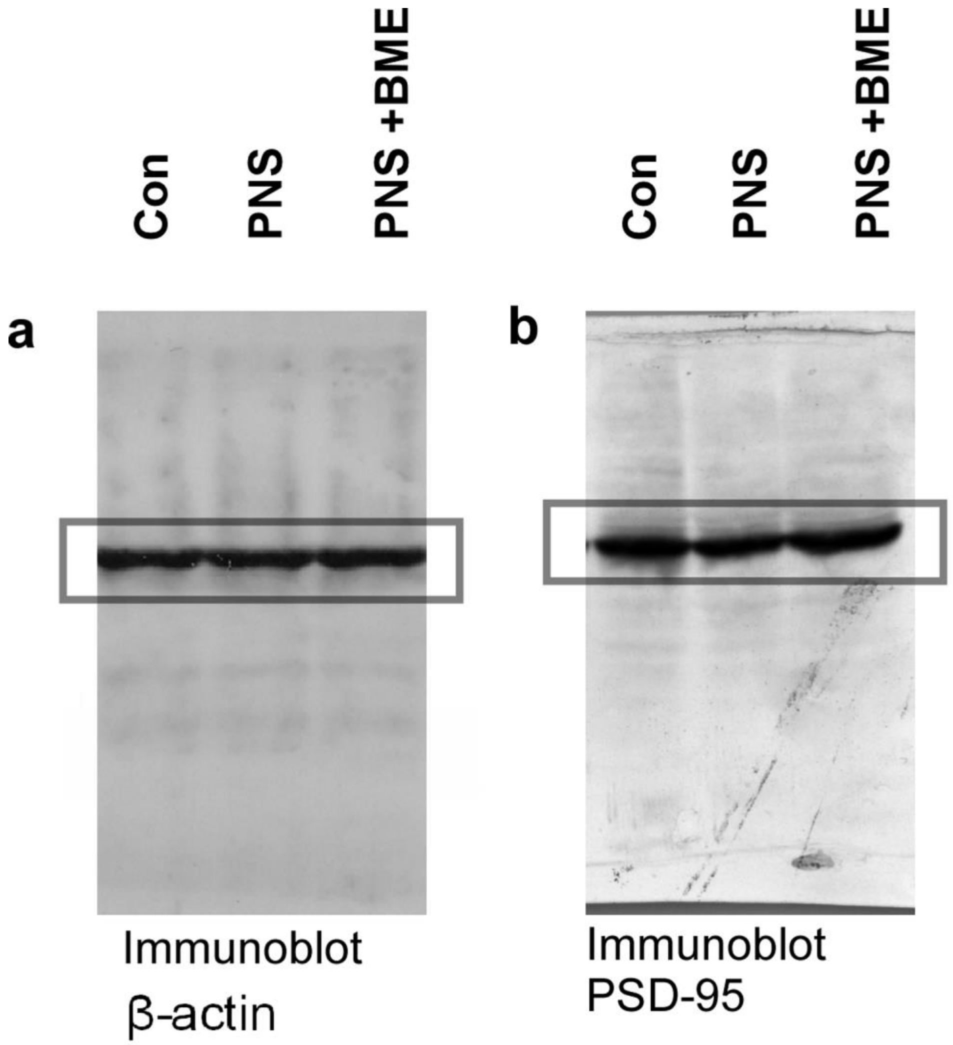

Supplementary Figure 9. Full immunoblot (uncropped) (a) β-actin (lower panel), (b) PSD-95 (upper panel), used for figure 11 in the manuscript. Gray rectangles are the images cropped from each blot that are shown in the manuscript and each lane representing experimental groups (Con: Control; PNS: prenatal stress group; PNS+ BME: prenatal stress group treated with *B. monnieri* extract).

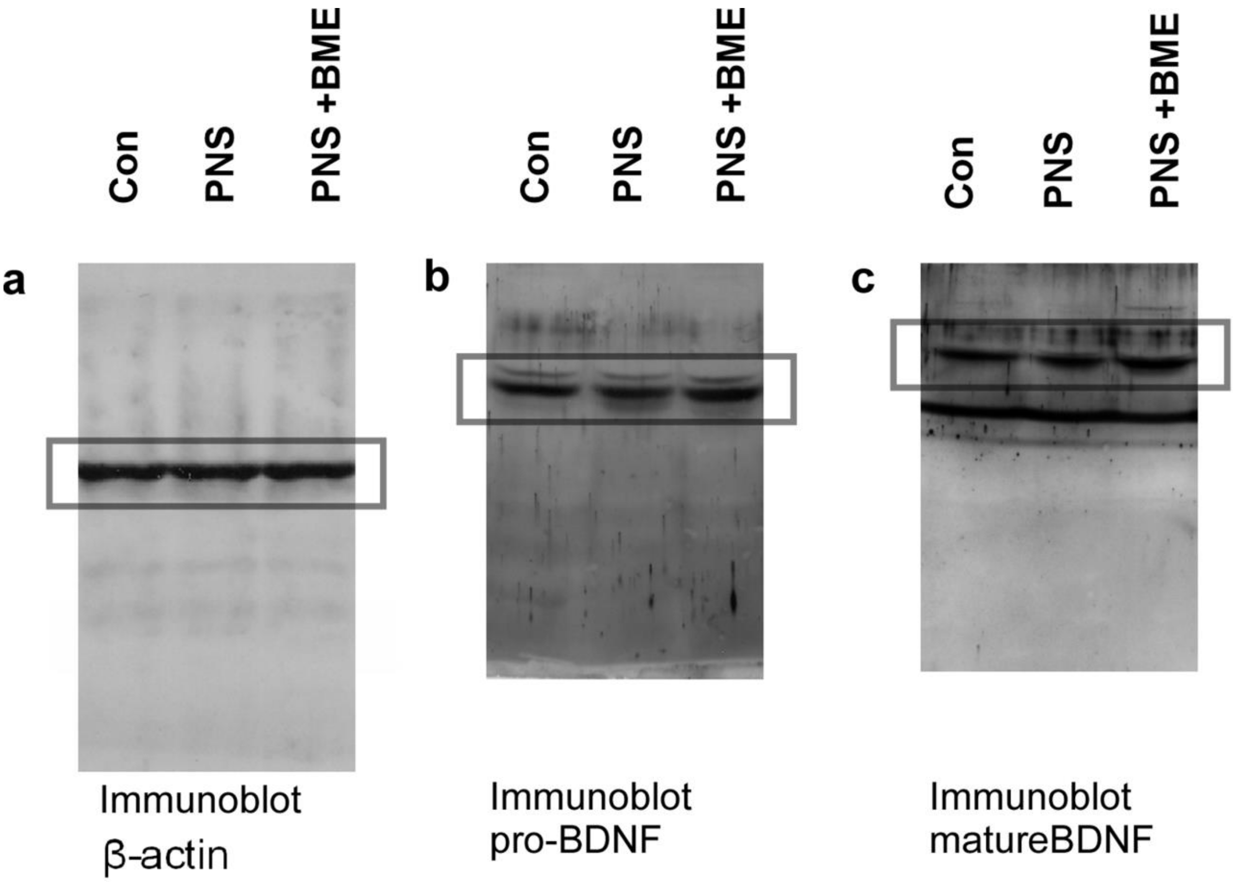

Supplementary Figure 10. Full immunoblot (uncropped) (a) β-actin (lower panel), (b) pro-BDNF (upper panel), (c) mature-BDNF (middle panel) used for figure 12 in the manuscript. Gray rectangles are the images cropped from each blot that are shown in the manuscript and each lane representing experimental groups (Con: Control; PNS: prenatal stress group; PNS+ BME: prenatal stress group treated with *B. monnieri* extract).

Representative westerblot image each band quantified trace values					
	Target Gene				
Figure 5		Control	Prenatal stressed	Prenatal Stressed +BME	
	GR	951575	1579669	737884	
		940100	1541813	723292	
		940100	1541813	723292	
	Actin	19847486	19253350	19292230	
		19554870	19196400	19420880	
		19014036	19544220	19788385	
Figure 6		Control	Prenatal stressed	Prenatal Stressed +BME	
	Casepase -3	589380	2054837	1018286	
		676476	2020226	1005205	
		662226	2015024	1018286	
	Bcl-2	10399266	9401760	10019869	
		10216854	9919520	10991401	
		10373994	9540160	10709250	
	Actin	1748805	1718652	1747008	
		1799175	1732544	1788864	
		1799175	1718652	1722912	
Figure 7		Control	Prenatal stressed	Prenatal Stressed +BME	
	SYP	10039250	7112290	9942690	
		10716375	7243472	9818803	
		10212625	7243472	9820648	
	SYT-1	4346750	2331472	4802680	
		4142875	2322632	4616596	
		4603875	2170168	4408372	
	Actin	19847486	19253350	19292230	
		19554870	19196400	19420880	
		19014036	19544220	19788385	

**Figure 9**		**Control**	**Prenatal stressed**	**Prenatal Stressed +BME**	
	**CaMKII**	12139968	9613296	11569350	
		12048543	9429552	11333140	
		12343488	9265104	11207820	
	**p-CaMKII**	1917755	606402	3641965	
		1862962	606402	3641965	
		1916185	538670	3261630	
	**T-Ng**	11184175	9530378	10018246	
		11184175	9353376	9787848	
		10977130	9353376	9789848	
	**p-Ng**	6511145	3306912	5598138	
		6613218	3423336	5700604	
		6943230	3500784	5700604	
	**Acting**	6864684	6014976	6528021	
		6610605	6438555	6626034	
		6732705	6438555	6773886	
**Figure 10**		**Control**	**Prenatal stressed**	**Prenatal Stressed +BME**	
	**NR2A**	7188020	3499020	4753140	
		7088340	3300640	4626300	
		7196280	3300640	4410280	
	**NR2B**	13250616	6963534	7991175	
		12617526	6411058	7667862	
		12954375	5849496	7426470	
	**Actin**	1761844	1758006	1775312	
		1714902	1737806	1737008	
		1728132	1721949	1764560	
		**Control**	**Prenatal stressed**	**Prenatal Stressed +BME**	
**Figure 11**	**PSD-95**	30860669	20238580	30036041	
		30757161	20112340	31230413	

		30353213	21612789	30132109	
	**Actin**	6864684	6014976	6528021	
		6610605	6438555	6626034	
		6732705	6438555	6773886	
		**Control**	**Prenatal stressed**	**Prenatal Stressed +BME**	
**Figure 12**					
	**pro-BDNF**	4122582	3663888	4801095	
		4861038	3593736	4308240	
		4559670	3371144	4502830	
	**mBDNF**	3287200	2505789	3243526	
		3103680	2382952	3205917	
		2933920	2308306	3119545	
	**Actin**	6864684	6014976	6528021	
		6610605	6438555	6626034	
		6732705	6438555	6773886	
