# Supplementary material for: Standardized Bacopa monnieri Extract Ameliorates Learning and Memory Impairments through Synaptic Protein, Neurogranin, Pro-and Mature BDNF Signaling, and HPA Axis in Prenatally Stressed Rat Offspring
Source: Antioxidants (Basel). 2020 Dec 4;9(12):1229. doi: 10.3390/antiox9121229 (PMC7761874; doi:10.3390/antiox9121229)
Supplement: Supplementary file 1 [file antioxidants-09-01229-s001.zip › Supplementary files/Supplementary data-1.pdf]

LMC

LUMEN MARKETING COMPANY

2, First Cross Street, Second Avenue, Ashok Nagar, Chennai 600083. India
Tel : 91 44 2485 3595 Fax : 91 44 4203 3176
E-mail : lumenmarketing@gmail.com web : www.lumen.co.in

FLOW CHART FOR MANUFACTURE OF CDRI 08

RAW MATERIAL

↓ Extraction with 70% Ethanol

EXTRACT

↓ Removal of solvent

DRY POWDER

↓ Treatment with 95% Ethanol

ETHANOL SOLUBLES

↓ Concentration and drying under vacuum

DRY FLAKES

↓ Pulverisation, Sieving and Packing

DRY POWDER EXTRACT

LUMEN MARKETING COMPANY CHENNAI 600 083 ★

For LUMEN MARKETING COMPANY

Authorised Signatory

CHEMILOIDS HPLC REPORT

C:\LabSolutions\Data\Project1\PROMINENCE-2 UV\DATA 2015\MAR 2015\BACMAR1603.lcd

Acquired by : Admin
Sample Name : Bacopa monnieri Extract Dry Powder
Sample ID : C15030294
Injection Volume : 20 uL
Data File Name : BACMAR1603.lcd
Method File Name : BACOPA (USP).lcm
Data Acquired : 3/16/2015 2:48:13 PM
Data Processed :1/9/2016 3:54:18 PM

<Chromatogram>

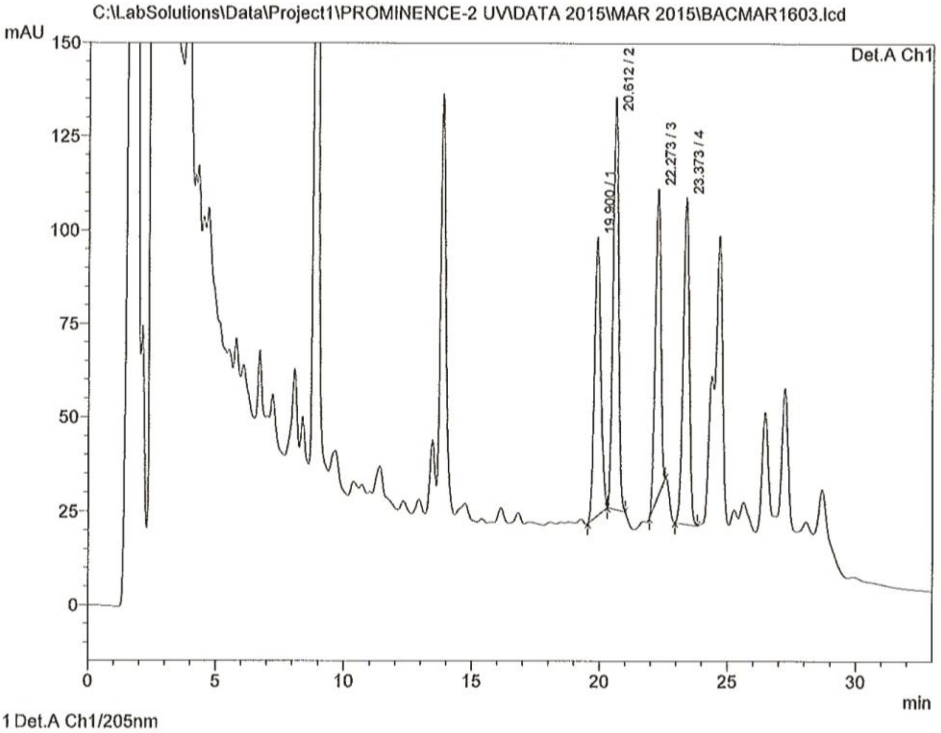

1 Det.A Ch1/205nm

Bacopa monnieri Extract Dry Powder

Batch No: C15030294

Peak No	Name of the Bacopaside	Retention time	Peak area	* Absolute Percentage of extract
1	Bacopaside A3	19.900	1286318	3.09%
2	Bacopacoside II	20.612	1653575	3.13%
3	Bacopa saponin X	22.273	1237478	2.77%
4	Bacopasaponin C	23.373	1439917	2.64%
	Total Bacosides By HPLC Method			11.63%

* The analysis was done as USP method, but using working standard for individual bacopaside.

Laila

CHEMILOIDS

CERTIFICATE OF ANALYSIS

March 23, 2015

1. Name of the Product	:	Bacopa monnieri Extract Dry Powder	
2. Product Code	:	C/LMN/BAMO-01	
3. Batch Number	:	**C15030294**	
4. Mfg. Date	:	March, 2015	
5. Trade Name	:	Jalabrahmi	
6. Part of the Plant	:	Herb	
7. Herb to Product Ratio	:	12-15 :1	
8. Description	:	Brown Color Dry Powder	
9. Identification	:	HPTLC Finger Printing	
10. Extraction Medium	:	Alcohol	
		**Results**	**Limits**
11. Loss on Drying	:	0.18%	NMT 10%
12. Alcohol Soluble Extractives	:	98.37%	NLT 90% on d/b
13. Sulphated Ash	:	6.57%	NMT 10%
14. Assay (Bacosides)	:	62.18%	NLT 55% on d/b
By Spectrophotometric Method			
**Microbial Limits**			
15. Total Aerobic Count	:	1300 Cfu/gm	NMT 25,000 Cfu/gm
16. Yeast & Molds	:	60 Cfu/gm	NMT 100 Cfu/gm
17. E.coli	:	Absent	Negative
18. Salmonella	:	Absent	Negative

The sample COMPLIES with the above said Specifications

Analyzed by

Q.C. CHEMIST

Approved by

Q.C. MANAGER

ADM. OFF : 40-15-14, BRINDAVAN COLONY, VIJAYAWADA - 520 010, INDIA.
Tel : +91-866-6668001, 2473468, 2476561, Fax : +91-866-2475278, 6668006
e-mail : vjwlimpex@sify.com; vjwlimpex@sancharnet.in
