# Supplementary material for: Standardized Bacopa monnieri Extract Ameliorates Learning and Memory Impairments through Synaptic Protein, Neurogranin, Pro-and Mature BDNF Signaling, and HPA Axis in Prenatally Stressed Rat Offspring
Source: Antioxidants (Basel). 2020 Dec 4;9(12):1229. doi: 10.3390/antiox9121229 (PMC7761874; doi:10.3390/antiox9121229)
Supplement: Supplementary file 1 [file antioxidants-09-01229-s001.zip › Supplementary files/Supplementary Informations.pdf]

Supplementary Informations

Standardized *Bacopa monnieri* extract ameliorates learning and memory impairments through synaptic protein, Neurogranin, pro-and mature BDNF signaling and HPA axis in prenatally stressed rat offspring

Karunanithi Sivasangari and Koilmani Emmanuvel Rajan*

Behavioural Neuroscience Laboratory, Department of Animal Science,

Bharathidasan University, Tiruchirappalli -620024, India

*Corresponding Author

Koilmani Emmanuvel Rajan

Behavioural Neuroscience Laboratory

Department of Animal Science,

Bharathidasan University, Tiruchirappalli -620024, India

Email: emmanuvel1972@yahoo.com

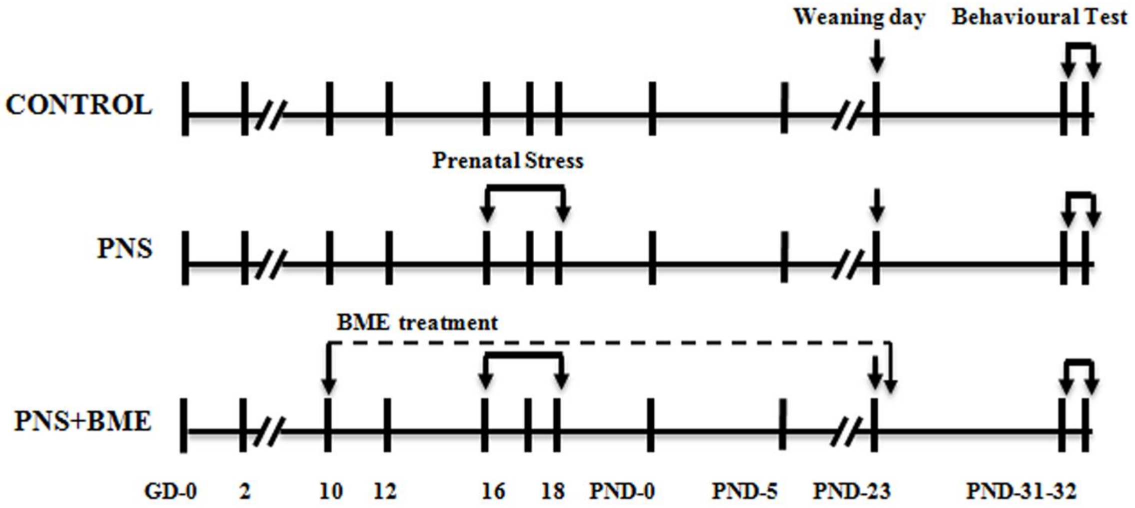

Supplementary Figure 1. Experimental timeline of events in the study, timeline showing the sequence of events and behavioural testing the animals underwent. Experiment initiated from gestational day (GD) - 0 and terminated after behavioural testing on postnatal day (PND)- 32. Three groups of animals were used: (i) control (CON); (ii) prenatal stress [PNS; received 0.5% gum acacia treatment (per-orally; p.o.)]; and (iii) prenatal stress+ treated with standardized extract of *Bacopa monnieri* (CDRI-08; mentioned as BME) [(PNS+BME + gum acacia (p.o.)] treatment. Except the control group, other two groups were subjected to PNS from GD-16 to 18. BME treated orally to the dams everyday (10.00 to 11.00 hr) from GD-10 to their pup's postnatal day (PND)-23 and to the pups from PND-15 to 30. All groups were sacrificed after behavioural testing for molecular analysis.

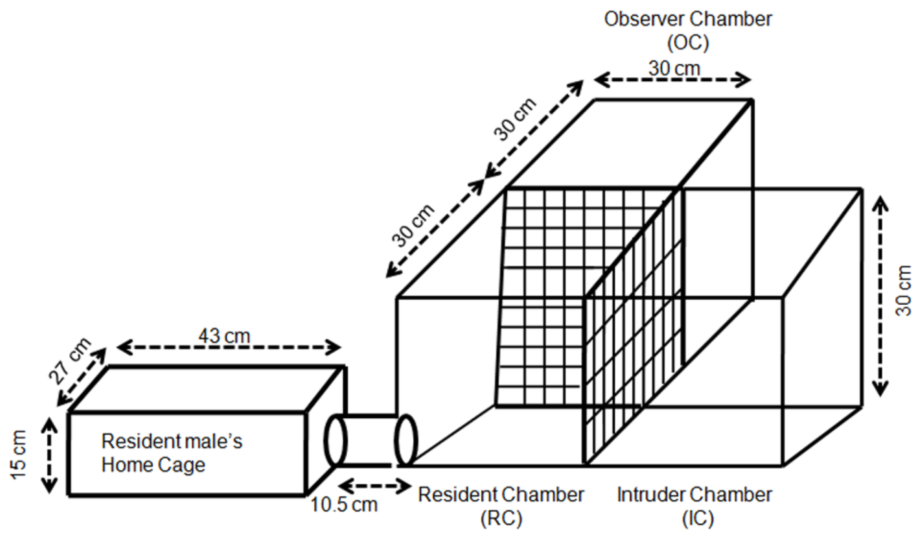

Supplementary Figure 2. Diagram showing the apparatus to induce gestational stress through social defeat observation.

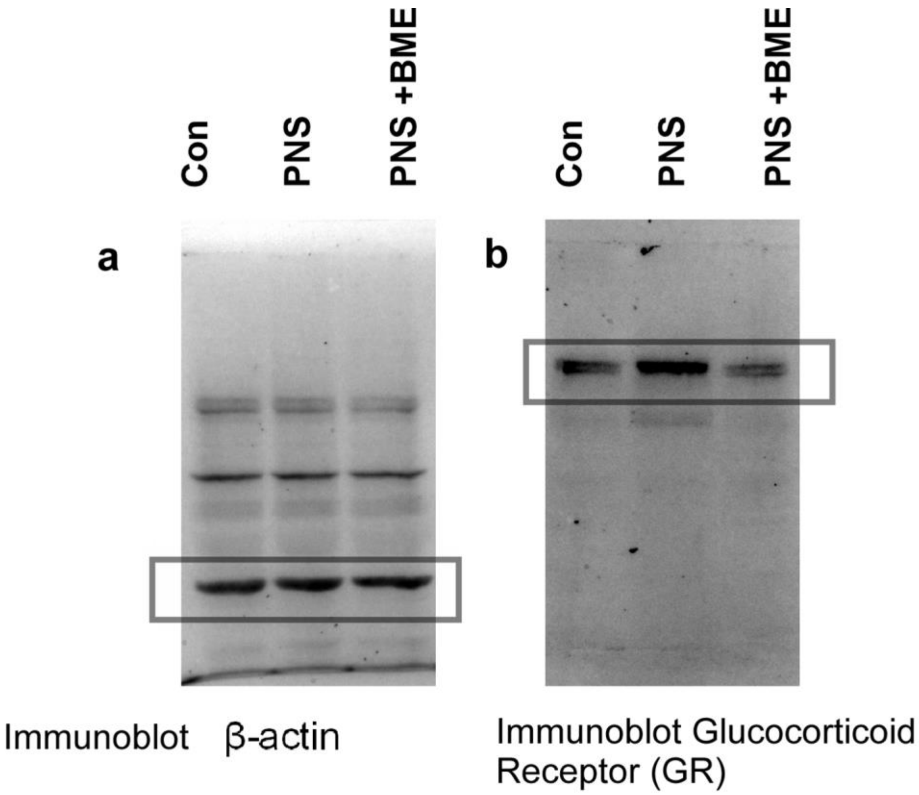

Supplementary Figure 3. Full immunoblot (uncropped) (a) β-actin (lower panel), (b) Glucocorticoid receptor (GR) (upper panel) used for figure 5 in the manuscript. Gray rectangles are the images cropped from each blot that are shown in the manuscript and each lane representing experimental groups (Con: Control; PNS: prenatal stress group; PNS+ BME: prenatal stress group treated with *B. monnieri* extract).

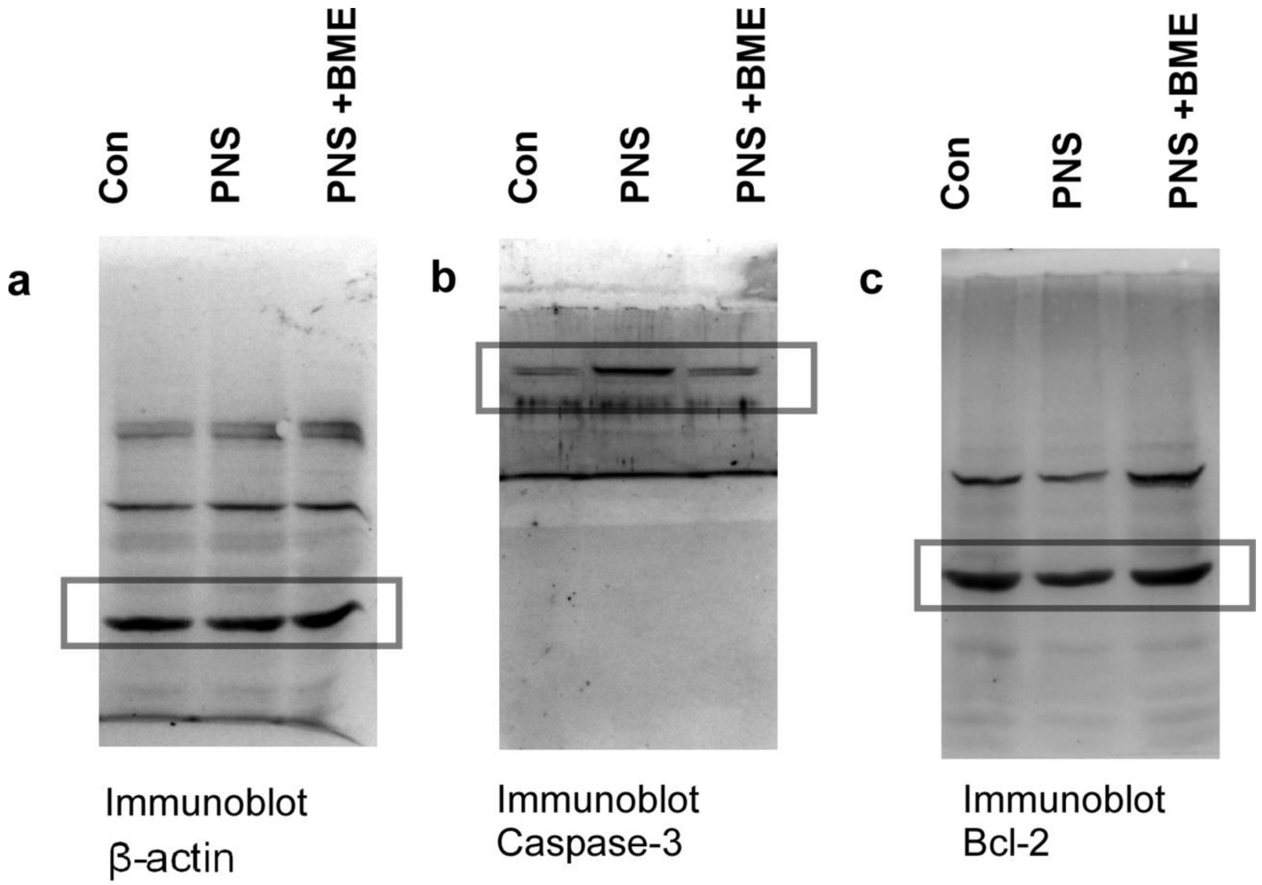

Supplementary Figure 4. Full immunoblot (uncropped) (a) β-actin (lower panel), (b) Caspase-3 (upper panel), (c) Bcl-2 (middle panel) used for figure 6 in the manuscript. Gray rectangles are the images cropped from each blot that are shown in the manuscript and each lane representing experimental groups (Con: Control; PNS: prenatal stress group; PNS+ BME: prenatal stress group treated with *B. monnieri* extract).

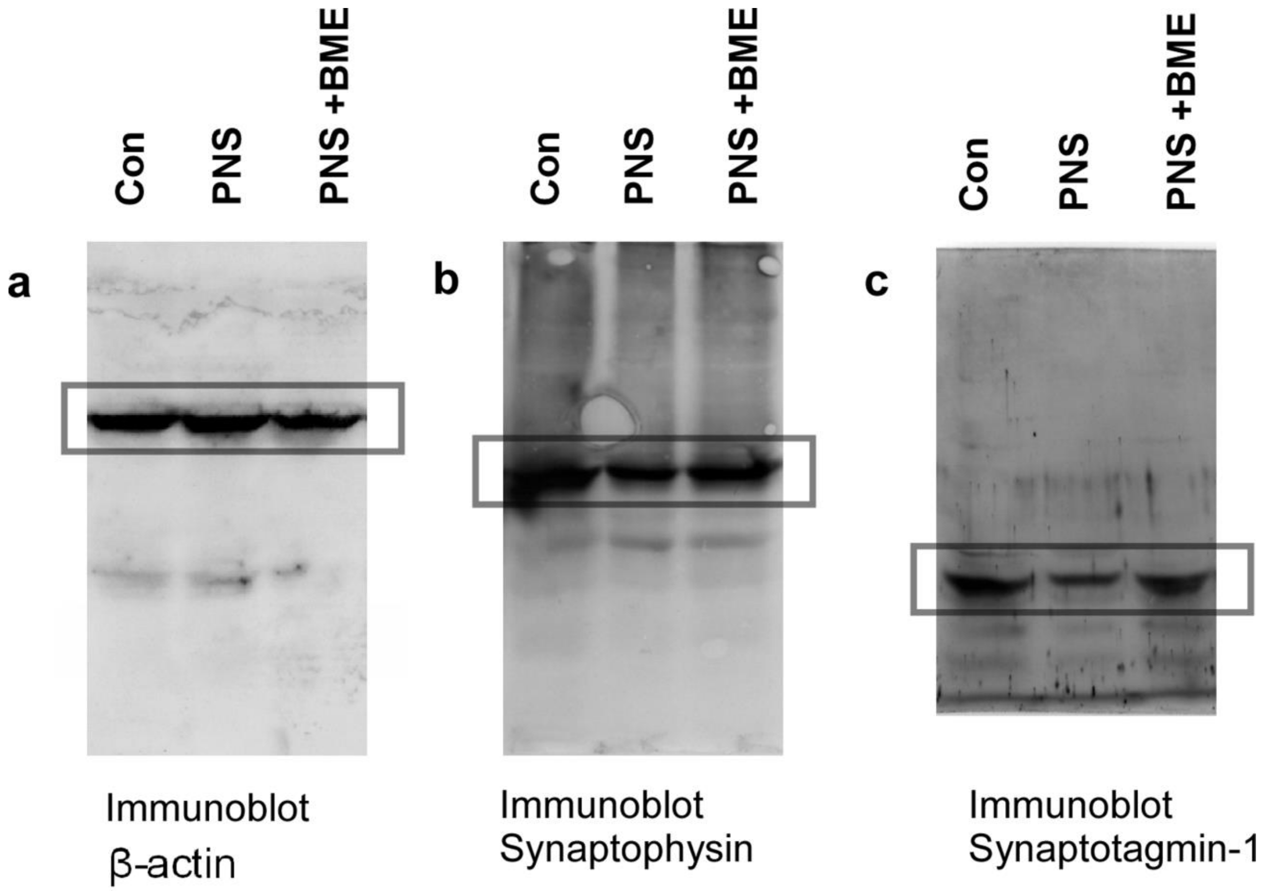

Supplementary Figure 5. Full immunoblot (uncropped) (a) β-actin (lower panel), (b) Synaptophysin (upper panel), (c) Synaptotagmine-1 (middle panel) used for figure 7 in the manuscript. Gray rectangles are the images cropped from each blot that are shown in the manuscript and each lane representing experimental groups (Con: Control; PNS: prenatal stress group; PNS+ BME: prenatal stress group treated with *B. monnieri* extract).

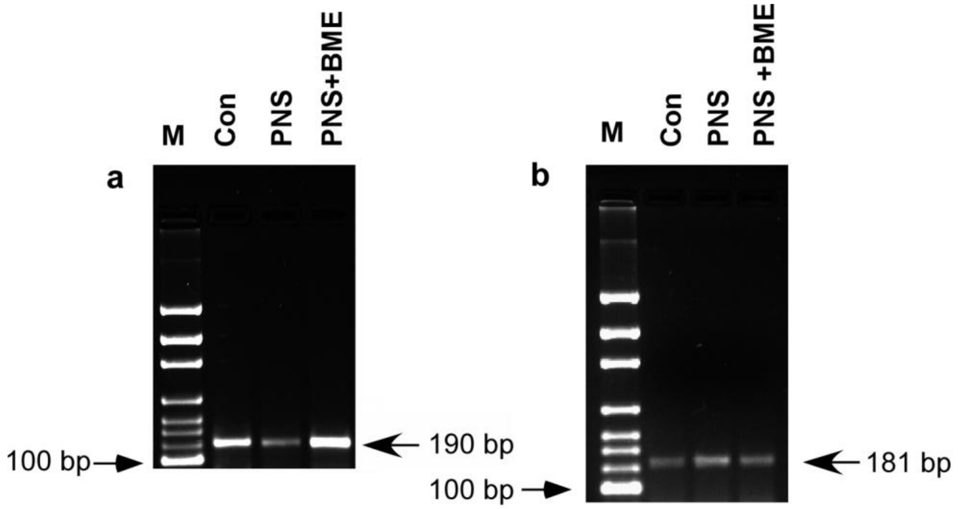

Supplementary Figure 6. Ethidium bromide stained agarose gel showing the mRNA level of (a) 5-HT1a, (b) 5-HT2c receptors amplified from experimental groups (Con: Control; PNS: prenatal stress; PNS+ BME: prenatal stressed treated with *B. monnieri* extract). Real-time PCR product is supporting for figure 8 in the manuscript.

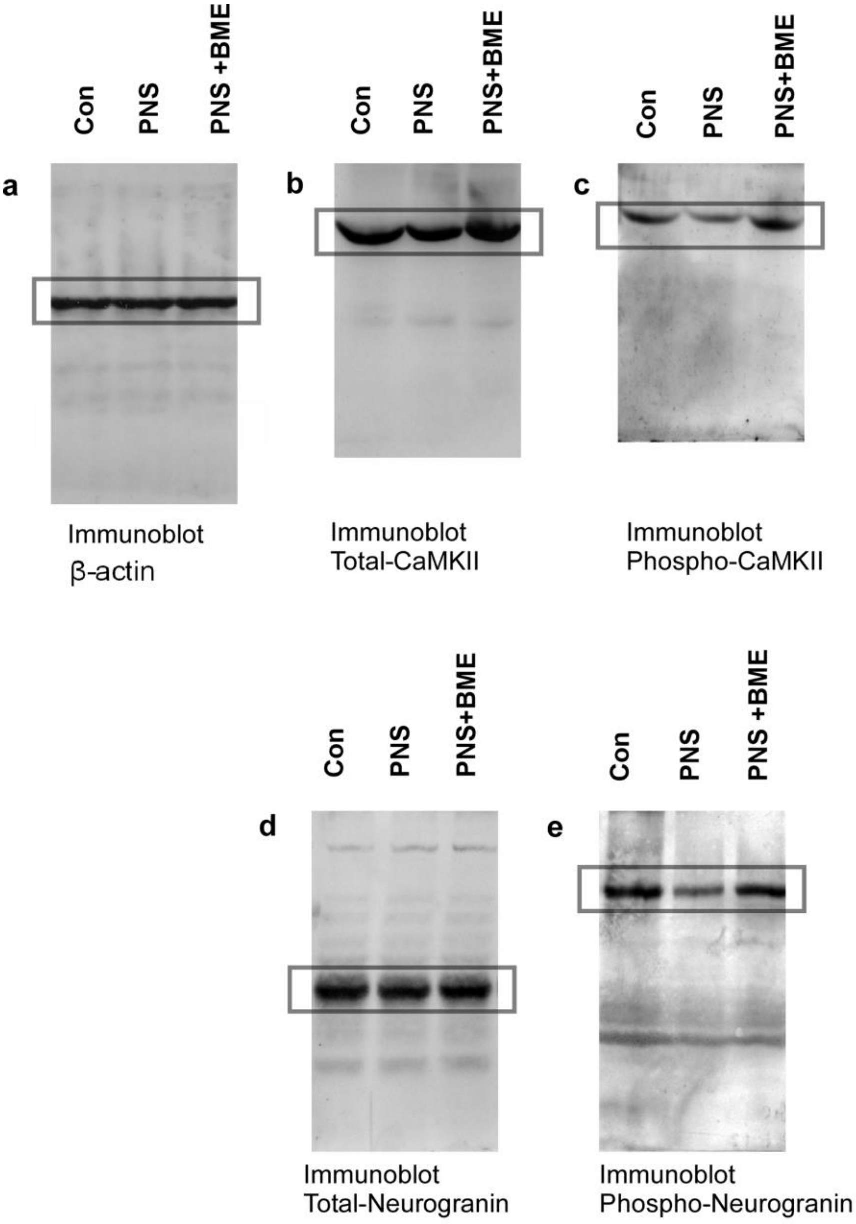

Supplementary Figure 7. Full immunoblot (uncropped) (a) β-actin (last panel), (b) total-CaMKII (first panel), (c) phosphorylated-CaMKII (second panel), (d) total-Neurogranin (third panel) and (e) phosphorylated-Neurogranin (fourth panel) used for figure 9 in the manuscript. Gray rectangles are the images cropped from each blot that are shown in the manuscript and each lane representing experimental groups (Con: Control; PNS: prenatal stress group; PNS+ BME: prenatal stress group treated with *B. monnieri* extract).

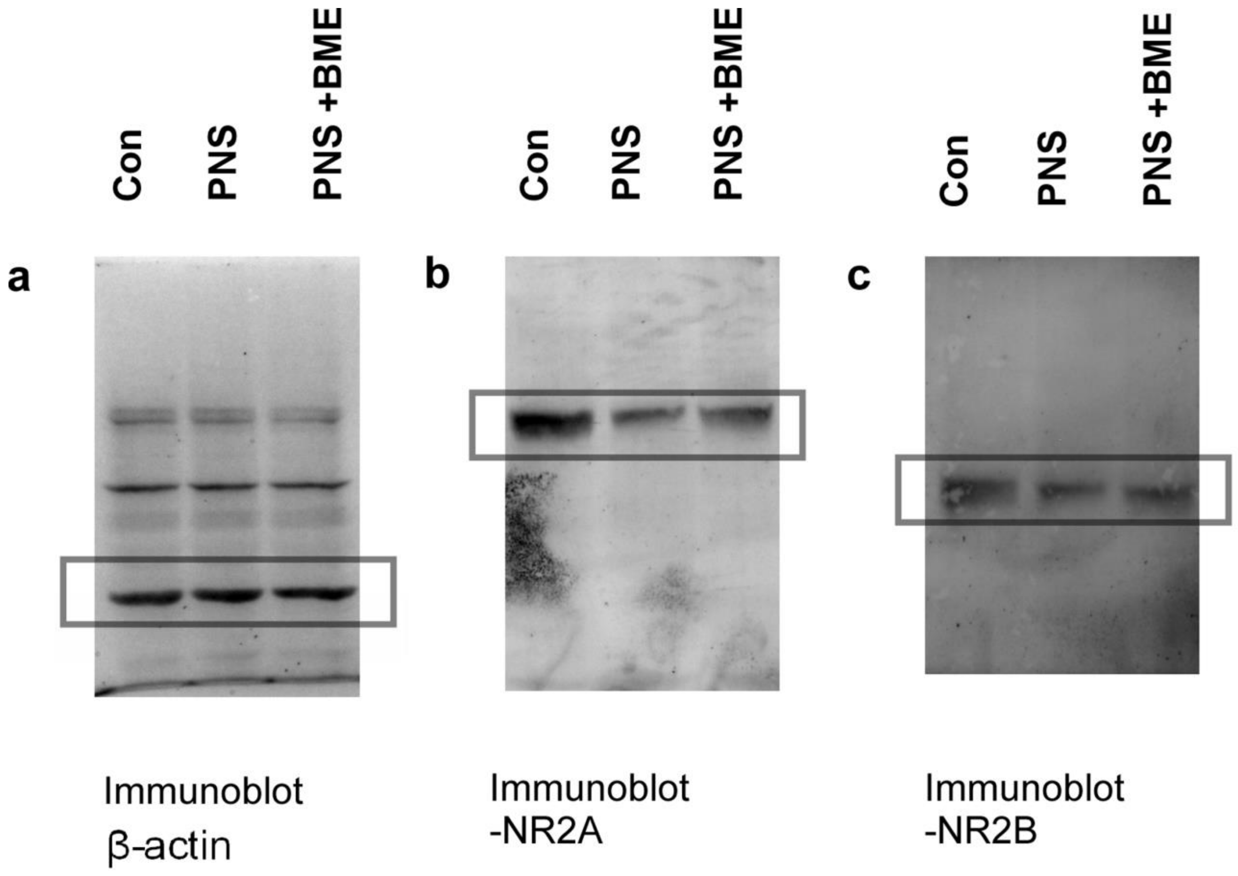

Supplementary Figure 8. Full immunoblot (uncropped) (a) β-actin (lower panel), (b) NR2A (upper panel), (c) NR2B (middle panel) used for figure 10 in the manuscript. Gray rectangles are the images cropped from each blot that are shown in the manuscript and each lane representing experimental groups (Con: Control; PNS: prenatal stress group; PNS+ BME: prenatal stress group treated with *B. monnieri* extract).

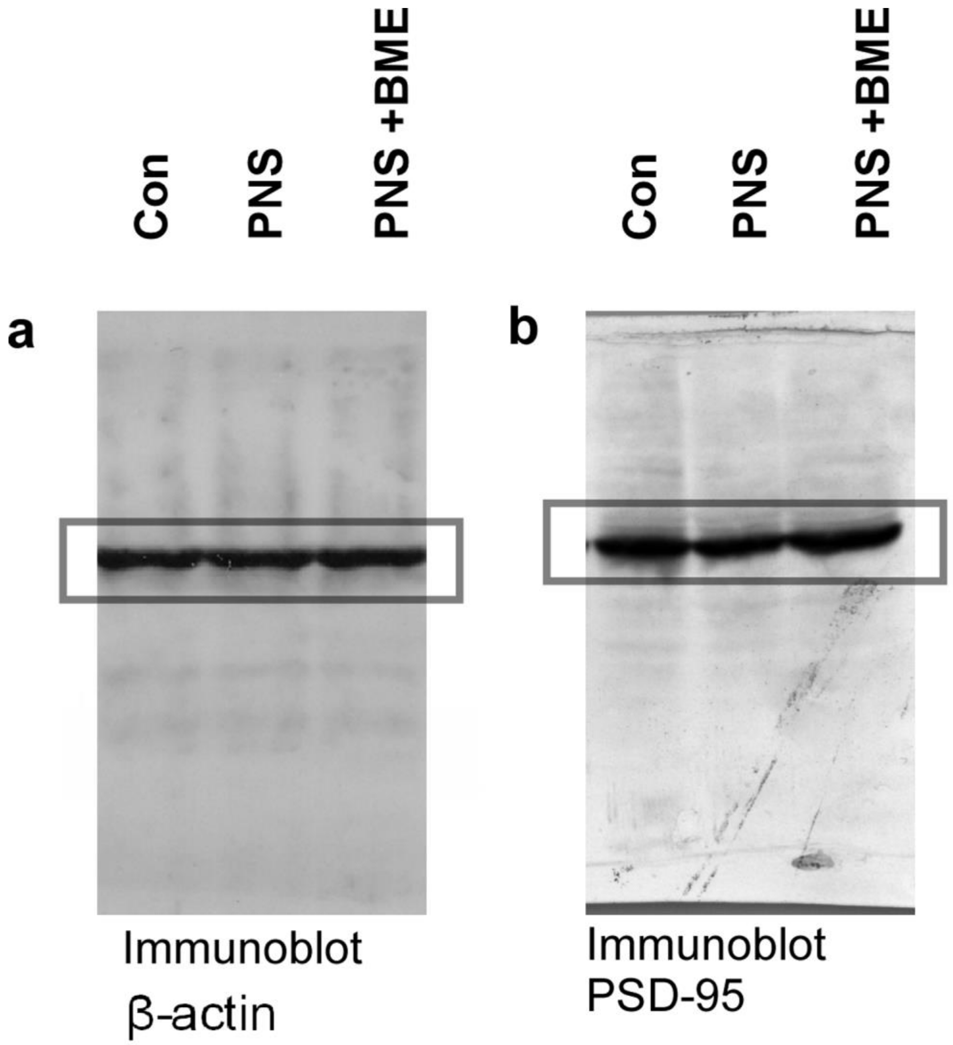

Supplementary Figure 9. Full immunoblot (uncropped) (a) β-actin (lower panel), (b) PSD-95 (upper panel), used for figure 11 in the manuscript. Gray rectangles are the images cropped from each blot that are shown in the manuscript and each lane representing experimental groups (Con: Control; PNS: prenatal stress group; PNS+ BME: prenatal stress group treated with *B. monnieri* extract).

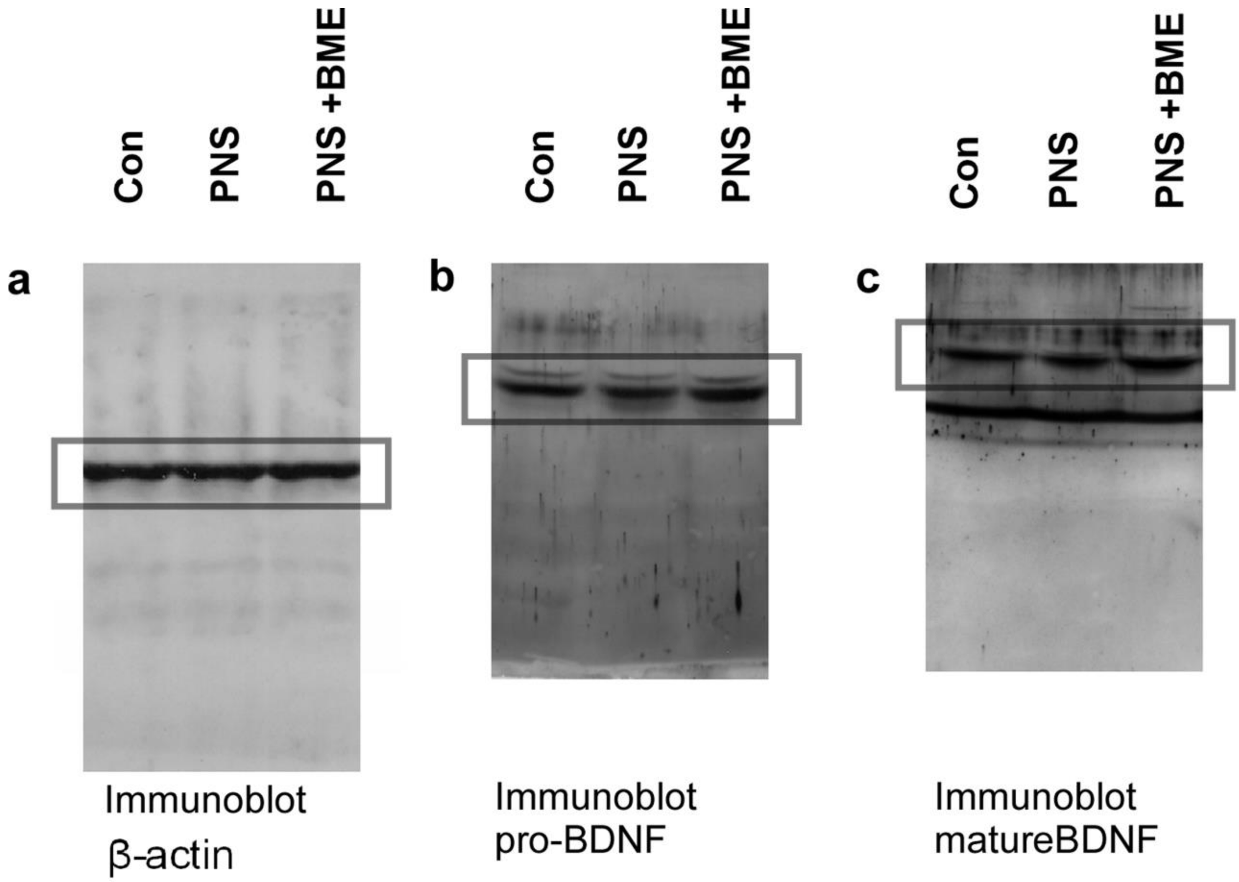

Supplementary Figure 10. Full immunoblot (uncropped) (a) β-actin (lower panel), (b) pro-BDNF (upper panel), (c) mature-BDNF (middle panel) used for figure 12 in the manuscript. Gray rectangles are the images cropped from each blot that are shown in the manuscript and each lane representing experimental groups (Con: Control; PNS: prenatal stress group; PNS+ BME: prenatal stress group treated with *B. monnieri* extract).
